# Design Concept of Metal Sulfide Photocatalyst for Efficient Photocatalytic Hydrogen Evolution

**DOI:** 10.1007/s40820-026-02111-0

**Published:** 2026-03-02

**Authors:** Qizhi Gao, Xinlong Zheng, Jiaxin Lin, Jiadi Zhai, Fan Yang, Xinjie Chen, Minghui Wang, Miaomiao Yang, Jing Li, Xiaodong Shi, Yonghao Xiao, Xinlong Tian, Yuhao Liu

**Affiliations:** State Key Laboratory of Tropic Ocean Engineering Materials and Materials Evaluation, School of Marine Technology and Equipment, School of Materials Science and Engineering, Hainan Provincial Key Lab of Fine Chem, School of Cyberspace Security (School of Cryptology), School of Physics and Optoelectronic Engineering, Hainan University, Haikou, 570228 People’s Republic of China

**Keywords:** Metal sulfide photocatalysts, Photocatalytic hydrogen evolution, Water splitting, Electronic dimensionality, Photocorrosion

## Abstract

Metal sulfide (MS) photocatalysts hold unique features of narrow-bandgap range, high light absorption coefficient, and suitable band structures, offering significant potential for efficient visible-light photocatalytic hydrogen evolution (PHE) via water splitting. However, the low electronic dimensionality of the traditional MS photocatalyst generally decreases the transfer and migration efficiency of the photogenerated charge carriers. In addition, severe intrinsic photocorrosion issue also severely reduces the photostability, hindering the practical application of PHE at scale. In this regard, the advanced design concept of MS photocatalysts, focusing on the high electronic dimensionality construction and efficient photocorrosion inhibition, is of great importance. This review firstly introduces the basic mechanisms of PHE, followed by an in-depth discussion of the fundamental distinction between structural dimensionality and electronic dimensionality, highlighting the superiority of 3D electronic connectivity in enabling isotropic charge migration and shallow defect states. Afterward, the MS photocatalysts with 3D electronic dimensionality and solutions to photocorrosion are systematically summarized, with a special emphasis on the emerging paradigm of advanced “controllable-photocorrosion,” which strategically utilizes the corrosion process to create active sites rather than merely suppressing it. Finally, the current unsolved challenges of MS photocatalysts are comprehensively discussed.

## Highlights

- Highlighting the essential 3D electronic dimensionality, isotropic orbital hybridization is shown to enhance charge carrier mobility in metal sulfide (MS) photocatalysts, overcoming structural dimensionality limits.
- A controllable-photocorrosion approach is developed to functionally harness corrosion, in situ generating catalytically active sulfur species that boost photocatalytic hydrogen evolution and structural durability.
- The intrinsic sulfur-coordination directionality synthesis method suppresses MS photocorrosion, offering a scalable stability enhancement, proved for CdS and ZnCdS systems.

## Graphical abstract

**Figure:**
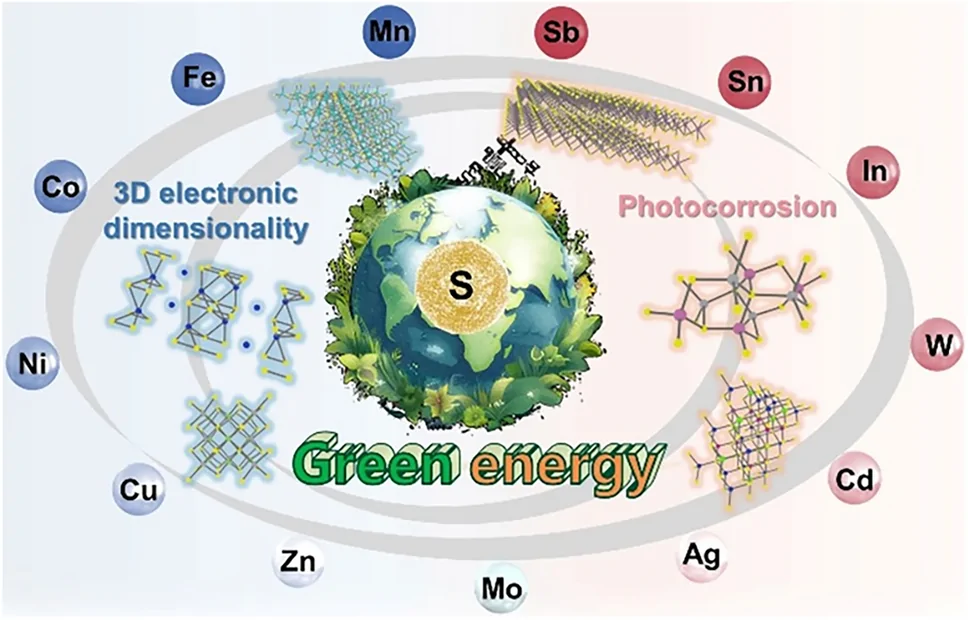

## Introduction

The prevailing reliance on traditional energy sources results in detrimental environmental impacts and severe energy security challenges [1–16]. The emerged hydrogen energy is a key candidate due to its promise of zero emissions and remarkably high energy density [17–24]. Among hydrogen production methods, photocatalytic hydrogen evolution (PHE) via water splitting stands out as a particularly promising approach for the direct and sustainable conversion of solar energy [25–30], relying on the exploration of semiconductor photocatalysts. Since the initial development of TiO_2_ for photoelectrochemical water splitting [31], the semiconductor-based photocatalysis has been established as a promising PHE pathway. This breakthrough spurred extensive research efforts aimed at enhancing the PHE efficiency of TiO_2_ [32–36]. At the same time, a wide array of other metal oxide (MO) photocatalysts, typically ZnO, CeO_2_, WO_3_, SnO_2_, and BiVO_4_, have been simultaneously investigated and applied in PHE technique [37–47]. However, the charge separation efficiency in most MO photocatalysts remains limited, largely due to the highly localized nature of O 2*p* states, which leads to a large effective mass of photogenerated holes. Moreover, the dominance of deeply lying of O 2*p* orbitals in the valence band (VB) results in wide bandgap [48]. In this case, PHE applications employing MO are generally restricted to ultraviolet (UV) light activation, which constitutes only about 4% of the solar spectrum. To achieve efficient PHE under visible light, the exploration and development of narrow-bandgap semiconductor photocatalysts are therefore imperative [49–51].

Metal sulfide (MS) semiconductor materials have garnered significant research attention as visible-light photocatalysts due to their inherently narrow bandgaps, which arise from S 3*p* orbitals, pronounced quantum size effects, and low carrier effective masses [52–59]. These attributes enable efficient solar spectrum utilization, extending light absorption from UV into the visible region, while their elevated conduction band (CB) potentials provide robust thermodynamic driving forces for hydrogen evolution reaction (HER) [60–62]. Typical examples including CdS, ZnCdS, ZnIn_2_S_4_, CuInS_2_, and Cu_2_ZnSnS_4_ (CZTS) represent a strategic research direction for achieving high-efficiency visible-light-driven PHE [63–68]. The approaches to improve the performance of MS photocatalysts, including morphology/structure engineering, elements doping, vacancy introduction, and heterojunction engineering, have been widely investigated [69–76], while the electronic dimensionality characteristics of MS photocatalysts have received little attention. Moreover, MS photocatalysts generally suffer from photocorrosion [77–80], which greatly hinders the long-term photostability. In this case, the design concept focusing on the electronic dimensionality and photocorrosion solutions is crucial for exploring the advanced MS photocatalysts to enable the scale-up PHE application.

**Fig. 1:**
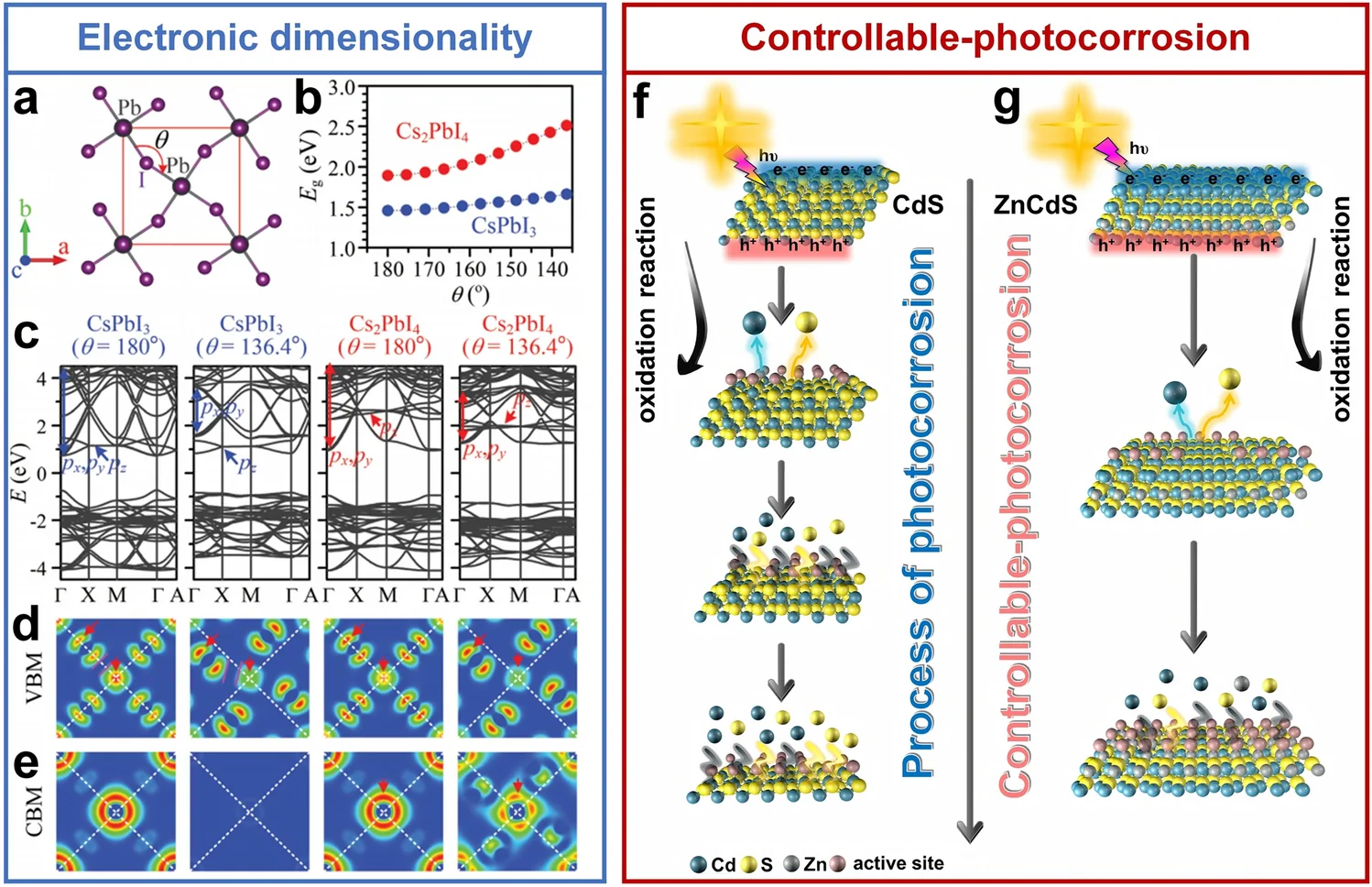
A visual exhibition of electronic dimensionality (blue part) and controllable-photocorrosion (red part). Fundamental concepts for advanced design of MS photocatalyst: **a** Schematic illustration of in-plane Pb–I–Pb bond angle (θ) variation in model 3D CsPbI_3_ and 2D Cs_2_PbI_4_ perovskites. **b** Calculated bandgaps increase as *θ* decreases, with 2D systems exhibiting greater sensitivity due to their lower electronic dimensionality. **c** Band structures and charge density distributions for the **d** VBM and **e** CBM under ideal (*θ* = 180°) and distorted (*θ* = 136.4°) configurations. Reduced orbital overlap along the inorganic framework diminishes electronic connectivity, effectively lowering the electronic dimensionality. This suppresses carrier dispersion and raises band edges, providing a conceptual link between structural distortion, electronic dimensionality, and band engineering in functional materials. Reproduced with the permission of Ref. [81]. Copyright 2017, Elsevier. Comparison of uncontrolled photocorrosion and engineered controllable-photocorrosion: **f** Schematic of the deleterious positive feedback loop in traditional MS (CdS as example): accumulation of photogenerated holes triggers uncontrolled sulfide oxidation (CdS + 2 h^+^ → Cd^2+^ + S), causing massive sulfur vacancy generation, structural degradation, and permanent activity loss. **g** Innovative “controllable-photocorrosion” paradigm based on ZnCdS solid solution in Ref. [84]. This self-limiting process involves: (i) The sacrificial sulfur-rich surface layer is selectively oxidized by holes, consuming the corrosive species and in situ generating catalytically active sulfur sites that enhance HER kinetics. (ii) The predefined corrosion front is autonomously halted upon reaching the underlying robust Zn–S layer, preventing bulk degradation. This transforms photocorrosion from a failure mechanism into a functional tool for performance augmentation and stability

The concept of “3D electronic dimensionality” transcends the conventional focus on structural dimensionality (0D, 1D, 2D, 3D). The structural dimensionality describes the geometric morphology, whereas electronic dimensionality defines the spatial connectivity of atomic orbitals constituting the band edges. A material can possess a 3D crystal structure but suffer from low electronic dimensionality due to directional orbital confinement, leading to the low charge migration efficiency. True 3D electronic dimensionality, characterized by isotropic orbital hybridization in all directions, is the key determinant for low effective mass, high carrier mobility, and benign defect properties [81–83]. At the same time, the controllable-photocorrosion strategy represents a paradigm shift from conventional corrosion suppression. Traditional approaches for solving photocorrosion, such as cocatalyst deposition or heterojunction construction, aim to passively prevent the oxidation of S^2−^ ions by extracting the photogenerated holes [63]. In contrast, the controllable-photocorrosion actively engineers the material to harness the photocorrosion process. The initial, controlled photocorrosion sacrificially consumes holes and, more importantly, in situ generates highly active sulfur species that boost the HER, while a robust subsurface layer halts further destruction. This transforms a detrimental process into a self-optimizing, functional mechanism for enhanced and stable performance. The visual contrast of 3D electronic dimensionality with lower dimensions and the corresponding illustration of controllable-photocorrosion mechanism are shown in Fig. 1. Therefore, on the basis of the construction of 3D electronic dimensionality, the substantial inhibition or fundamental solution to the photocorrosion issue of MS photocatalysts is also crucial.

In parallel with the increasing research efforts on MS photocatalysts, several representative reviews have focused on the photocatalytic applications. Typically, Chandrasekaran et al. comprehensively summarize controlled synthesis, property modulation, and applications of MS nanocrystals in electrocatalytic, PHE, and photoelectrochemical water splitting, highlighting structure–performance relationships and future scientific research directions [85]. Furthermore, considerable attention has also been given to emerging MS photocatalysts such as CuInS_2_, ZnIn_2_S_4_, ZnCdS, etc., which have been extensively reviewed for their broad PHE application [86–90]. Despite these notable reviews, a systematic overview that addresses both electronic dimensionality and innovative strategies for mitigating photocorrosion remains lacking. Given the key roles of 3D electronic dimensionality and photostability, this review highlights the recent advances of MS photocatalysts exhibiting 3D electronic dimensionality and effective resistance to the photocorrosion. The basic mechanism of PHE via water splitting is firstly introduced, and thereafter, the exploration of MS photocatalysts in PHE applications is concisely summarized. Subsequently, the design concept of 3D electronic dimensionality and photocorrosion solution of MS photocatalysts are provided, and the explored advanced MS photocatalysts with 3D electronic dimensionality and promising photocorrosion solution features are comprehensively discussed. Particularly, the evolution from lower (0D/1D/2D) to three-dimensional (3D) electronic connectivity represents a critical pathway to overcome the charge separation/migration and stability bottlenecks in next-generation MS photocatalysts. Finally, the current challenges and future research directions for advanced MS photocatalysts are presented, which may inspire the development of more suitable materials for the PHE.

## Fundamental Mechanisms and Core Concepts

### Mechanism of PHE via Water Splitting

**Fig. 2:**
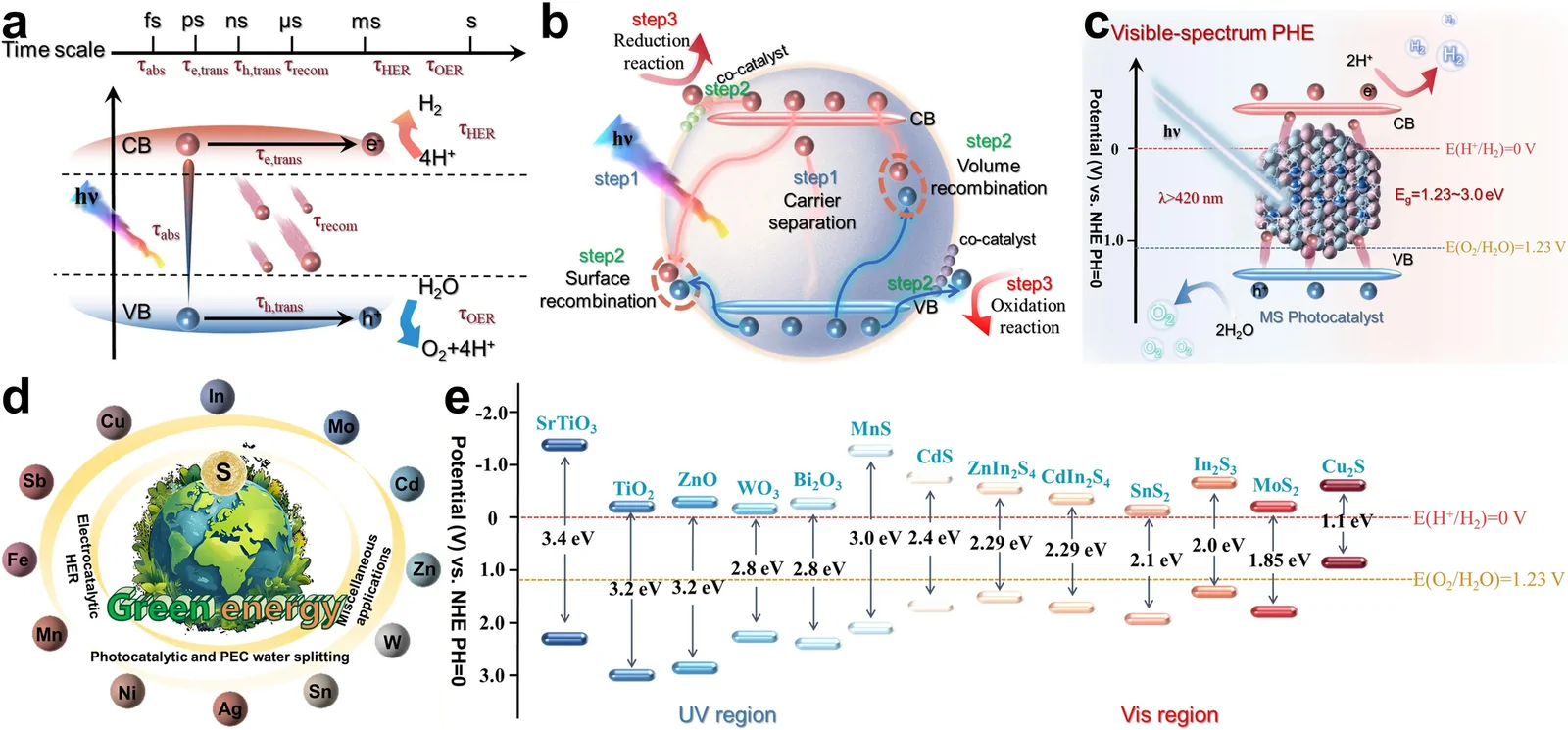
Basic mechanism illustration of PHE. **a** Photogenerated charge transfer behaviors and the corresponding consuming time. Reproduced with the permission of Ref. [25] Copyright 2015, Springer. **b** Charge migration behaviors during PHE process. **c** Schematic illustration of water splitting over semiconductor photocatalysts. The corresponding overall mechanistic content includes the steps of (i) adsorption of photon leading to the exciton state; (ii) photogenerated charges’ separation and migration; (iii) surface reduction in protons for HER and oxidation of water for OER. **d** Harnessing MS in the realm of renewable energy application utilizations. **e** Energy band potentials of MS photocatalyst under extensive investigation for PHE applications. Reproduced with the permission of Ref. [107] Copyright 2022, Wiley

Photocatalytic water splitting is a process involving the decomposition of water into hydrogen and oxygen [91–98], which necessitates incident photons with energies exceeding the thermodynamic threshold of 1.23 eV to generate the charge carriers (electron−hole pairs) under light irradiation. Concurrently, harnessing visible-light radiation for efficient PHE mandates a semiconductor band gap predominantly below 3.0 eV. Thermodynamically, water splitting is associated with a significant positive Gibbs free energy (Δ*G*_H*_ = 237 kJ mol^−1^) change, rendering it a highly endergonic process [64]. This substantial energy barrier constitutes a fundamental limitation for PHE approaches. PHE via water splitting comprises three pivotal phases that govern overall system efficiency [25, 99]: (i) Semiconductor photocatalysts initiate energy conversion by ultrafast generation of electron−hole pairs upon photon absorption (Fig. 2a). (ii) Segregated electrons and holes migrate to respective cocatalysts, driving HER and oxygen evolution reaction (OER) (Fig. 2b). Semiconductor photocatalysts require appropriate band potentials to drive HER and OER, while effective charge management is crucial to minimize major energy loss from recombination.

### Scientific Principle of Electronic Dimensionality

The realization of efficient PHE requests semiconductors with optimal photoelectric properties, most critically a bandgap tailored for visible-light-spectrum absorption (1.23–3.0 eV) to maximize solar energy utilization (Fig. 2c) [100–106]. However, beyond the bandgap, the efficiency of charge migration, a process pivotal to the second phase of PHE, is also fundamentally governed by less-discussed but paramount material’s feature: electronic dimensionality. Specifically, the electronic dimensionality surpasses the conventional classification based on the structural dimensionality, including 0D, 1D, 2D, 3D structures. It quantitatively describes the spatial connectivity and overlap of the atomic orbitals that constitute the VB maximum (VBM) and CB minimum (CBM). High electronic dimensionality, ideally approaching 3D connectivity feature, arises from extensive orbital hybridization along all the crystallographic directions. This results in isotropic band dispersion, low effective masses for both photogenerated electrons and holes, and high carriers’ mobility, enabling efficient and directionally unbiased charge migration to surface reaction sites. On the contrary, semiconductor materials with low electronic dimensionality (2D, 1D, and 0D) suffer from directional orbital confinement. It can lead to the anisotropic carrier transport, pronounced band tailing, and the formation of deep-level defect states that act as recombination centers, severely compromising the PHE efficiency. Therefore, the electronic dimensionality of semiconductor photocatalyst is significantly different from its traditional structural dimensionality due to the factors like specific chemical bonding or lattice distortions that impede orbital overlap. The design of advanced photocatalysts should prioritize achieving 3D electronic connectivity within the crystal structure. This ensures delocalized band edges, minimizes the formation of deleterious deep traps, and facilitates rapid charge separation/migration, forming the electronic foundation for high-performance PHE.

### Intrinsic Challenge of MS Photocatalysts: Photocorrosion

On the basis of achieving suitable bandgaps and efficient charge separation/migration, the long-term scale-up application of semiconductor photocatalysts is critically hindered by their inherent instability under light irradiation conditions, primarily due to the photocorrosion issue of MSs [108–114]. This degradation mechanism is an electrochemical corrosion process accelerated by the light irradiation during PHE. The core reaction involves the oxidation of lattice S^2−^ anions by photogenerated holes accumulated at the surface of MS photocatalysts, leading to irreversible structural destruction, generation of metal cations, formation of element sulfur, and a consequent rapid decay in PHE activity. The thermodynamic tendency for this chemical reaction is intrinsically linked to the easily destruction of the metal–sulfur bond during PHE process. Thus, the photocorrosion issue represents a fundamental stability challenge that is decoupled from, and often intensifies, limitations in charge dynamics. Only enhancing the charge separation does not inherently resolve this thermodynamic instability. Therefore, addressing photocorrosion issue requires dedicated strategies that either kinetically inhibit the oxidation of S^2−^ or thermodynamically redirect photogenerated holes toward alternative, productive reaction pathways before they can attack the lattice.

## Evolution of MS Photocatalysts: From Binary to Multinary Systems

Guided by the discussion of the fundamental requirements mentioned above, including visible-light absorption, efficient charge separation/migration, and operational photostability, the exploration progress of MS photocatalysts has undergone a significant evolution. This progression has been marked by strategic material design aimed at balancing these competing properties. The exploration began with the binary MS (BMS) such as CdS, MoS_2_, PbS, ZnS, etc., owing to their structural simplicity and facile synthesis processes [107, 115–119]. These BMSs demonstrated the initial promise of MS for efficient visible-light PHE. However, their practical application revealed intrinsic limitations, which has been comprehensively summarized by Zheng et al. [120]: CdS suffers from serious photocorrosion, MoS_2_ exhibits phase instability, and materials like CuS face issues with lattice mismatches in composite systems.

**Fig. 3:**
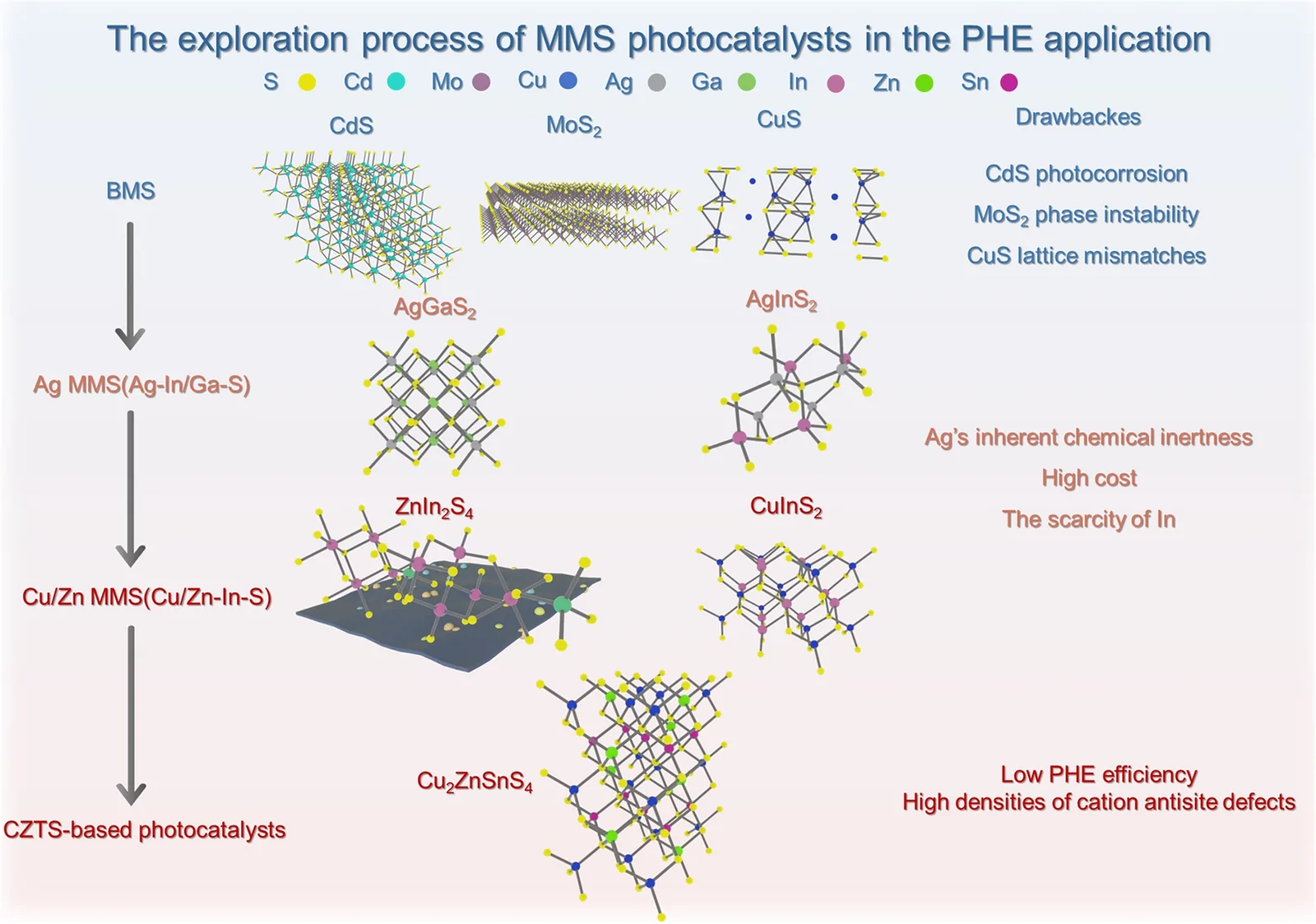
Major exploration process of MMS photocatalysts in PHE application

To overcome the intrinsic drawbacks, research advanced toward multinary MS (MMS), which offer enhanced chemical robustness, versatile elemental configurations, and tunable band structures [121–129]. Particularly noteworthy are MMS comprising group XI − XIII elements (Cu/Ag/Zn − In/Ga − S), which demonstrate exceptional solar energy harvesting capabilities, optimal bandgap energetics, and eco-compatibility, positioning them as premier platforms for efficient PHE (Fig. 2d, e) [130]. Figure 3 comprehensively summarizes the exploration process of MMS photocatalysts in the PHE application. Specifically, pioneering investigations focused on silver-based MMS photocatalysts (Ag-MMS), such as AgGaS_2_, AgInS_2_, AgBiS_2_, and AgIn_5_S_8_, leveraging their eco-compatible constituents and exceptional photostability within optimal bandgap ranges for solar harvesting. Nevertheless, the thermodynamic constraints are imposed by the inherent chemical inertness of silver, hindering the fabrication of monodisperse phase-pure nanostructures.

**Fig. 4:**
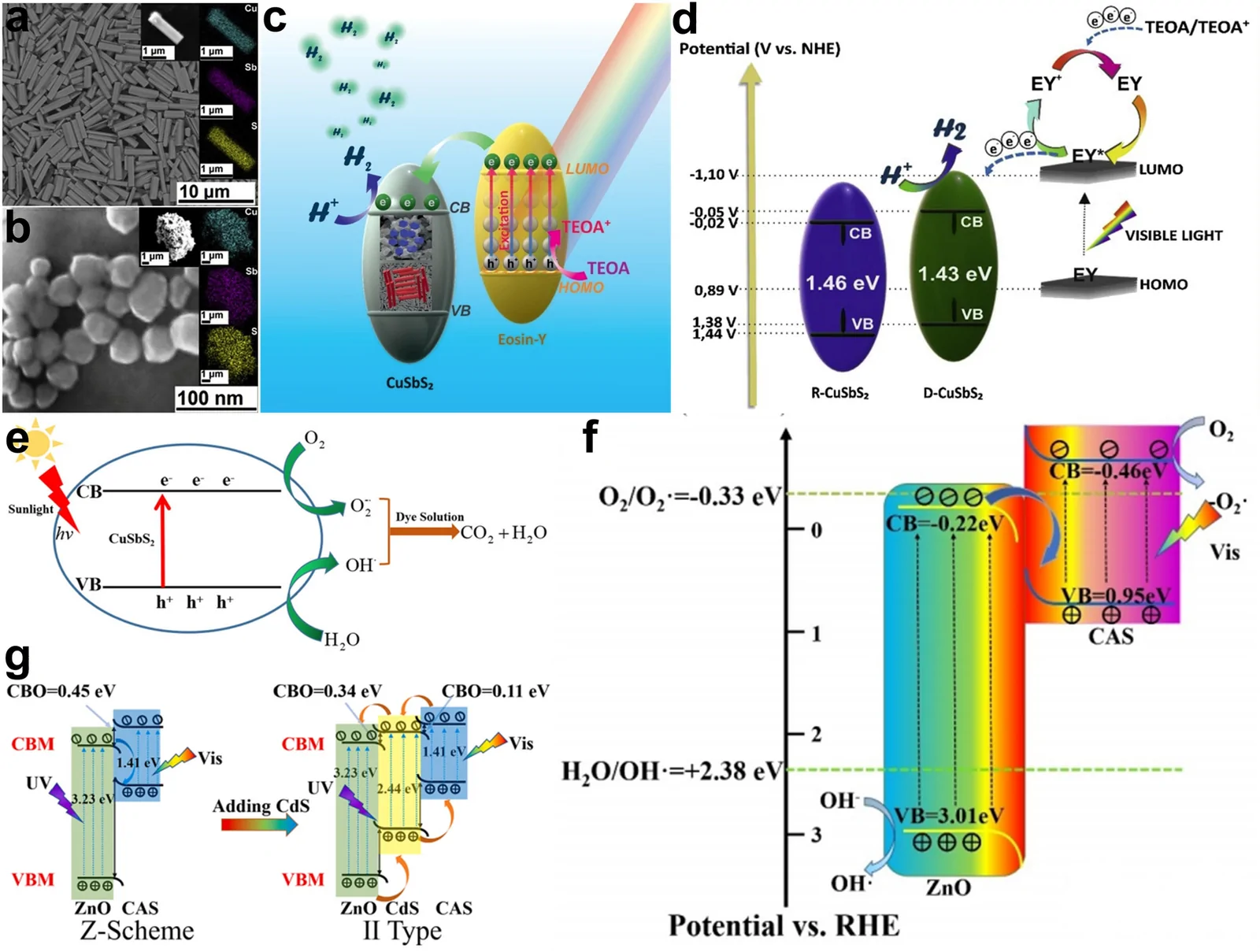
**a, b** SEM image of synthesized CuSbS_2_ photocatalyst with nanorod and nanodot morphology. **c, d** Schematic representation of the PHE mechanism mediated by CuSbS_2_ photocatalyst. Reproduced with the permission of Ref. [139] Copyright 2020, Elsevier. **e** Operando mechanistic pathways of PD for RhB via microwave-synthesized CuSbS_2_ nanostructures. Reproduced with the permission of Ref. [140] Copyright 2020, Springer. Interfacial charge migration modifications of CuSbS_2_-based photocatalysts: **f** Charge carrier dynamics of photogenerated electron−hole pairs in ZnO/CuSbS_2_ heterostructures and formation of reactive oxygen species under solar illumination. Reproduced with the permission of Ref. [142] Copyright 2022, Wiley. **g** Energy band alignment and carrier transport dynamics in CuSbS_2_ before and after CdS incorporation, illustrating. Reproduced with the permission of Ref. [141] Copyright 2023, Elsevier

**Fig. 5:**
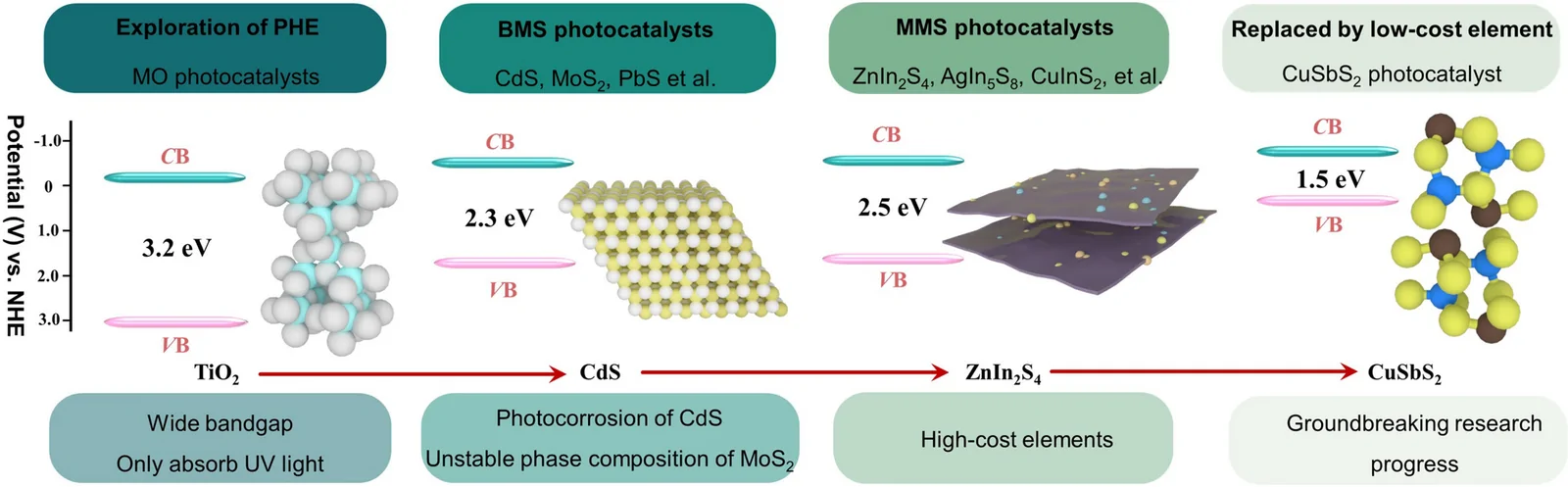
Exploration route of MS photocatalysts: MO → BMS → MMS → CuSbS_2_. The examples of each type of photocatalysts listed in the middle are: TiO_2_ (MO), CdS (BMS), and ZnIn_2_S_4_ (MMS)

Copper-/zinc-based MMS systems offer simplified synthesis and are promising for enhanced PHE [63]. Among these, CuInS_2_ and ZnIn_2_S_4_ have attracted significant interest due to their facile synthesis, suitable bandgaps (~ 1.5 and ~ 2.5 eV), and eco-friendly composition [131–133]. Notably, ZnIn_2_S_4_ has shown particular promise for efficient PHE [134–137]. To reduce reliance on expensive indium, earth-abundant alternatives such as antimony have been explored, leading to materials like CuSbS_2_ [64, 138]. This cost-effective photocatalyst not only addresses material limitations but also exhibits considerable PHE potential. Sarilmaz et al. first reported microrod and nanodot CuSbS_2_ for hydrogen production (Fig. 4a–d) [139]. Subsequent studies developed modified CuSbS_2_ and heterojunctions (CuSbS_2_/CdS and CuSbS_2_/TiO_2_) for PHE and degradation of Rhodamine B (Fig. 4e–g) [140–143]. Therefore, the progression of MS photocatalysts can be summarized as BMS of CdS, MoS_2_, PbS, CuS, etc. → MMS of Cu/Ag/Zn − In/Ga − S with high-cost element → MMS of CuSbS_2_ with low-cost elements (Fig. 5). Noticeably, MS photocatalysts generally exhibit excellent PHE performance, benefiting from their suitable electronic band structures and strong light harvesting capabilities. However, the VB potentials and interfacial kinetics of many MS systems make direct water oxidation and oxygen evolution difficult, and the photogenerated holes tend to oxidize lattice S^2−^ species, leading to severe photocorrosion [144–149]. To circumvent this issue, the sacrificial reagents, representatively Na_2_S/Na_2_SO_3_, lactic acid, and triethanolamine, are commonly introduced to selectively consume the photogenerated holes with low oxidability, thereby facilitating the reduction half-reaction for hydrogen evolution over the MS photocatalysts.

## Advanced Design Concept of MS Photocatalysts: 3D Electronic Dimensionality and Photocorrosion Mitigation

Throughout these investigations, the structural dimensionality, spanning from 0 to 3D architectures, has emerged as a governing factor governing PHE efficiency in MS systems [150–154]. This assertion is partially premised upon the fundamental principle that during PHE process, photogenerated electrons must efficiently migrate to active surface sites before recombination with photogenerated holes [155]. Given the stochastic grain orientation inherent in the semiconductor photocatalysts, isotropic charge migration characteristics significantly enhance the probability of electron migration to the photocatalytic active sites [156]. This section delineates cutting-edge design concept for MS photocatalysts through rigorous analysis of electronic dimensionality, a fundamental descriptor of atomic orbital connectivity forming the CBM and VBM manifolds [81]. This framework elucidates critical photophysical properties including bandgap engineering, charge carrier mobility, and defect state energetics.

### Scientific Illustration of Electronic Dimensionality

Electronic dimensionality describes the degree of connectivity and overlap between the orbitals that form the VBM and CBM. This connectivity governs the spatial delocalization of the charge carriers, thereby directly influencing fundamental electronic properties such as band dispersion, effective mass, optical transition probabilities, and electronic isotropy. A higher electronic dimensionality, particularly 3D electronic network, effectively promotes enhanced carrier mobility and improved charge transport by facilitating overlapping orbital pathways. It is therefore widely recognized, through both theoretical and experimental studies, that materials exhibiting 3D electronic dimensionality generally offer superior potential for high-performance solar-to-energy conversion. Illustratively, many leading photovoltaic semiconductors, including Si, CdTe, GaAs, Cu(In,Ga)Se_2_, and perovskite, possess not only 3D crystal structures but 3D electronic connectivity [157–162], which underpins their excellent optoelectronic performance. Therefore, electronic dimensionality serves as a fundamental and predictive descriptor for understanding and tailoring the optoelectronic behavior of functional semiconductors [163]. Specifically, high electronic dimensionality, approaching 3D behavior, occurs when orbitals hybridize extensively along all crystallographic directions, enabling isotropic band dispersion, low effective carrier masses, and reduced defect-induced recombination. Conversely, low electronic dimensionality manifests as directional orbital confinement, resulting in anisotropic carrier mobility, band tailing, and deep defect states that act as nonradiative recombination centers. Crucially, electronic dimensionality may deviate significantly from structural dimensionality due to chemical substitution or lattice distortion that disrupt orbital overlap without altering the macroscopic crystal lattice topology.

**Fig. 6:**
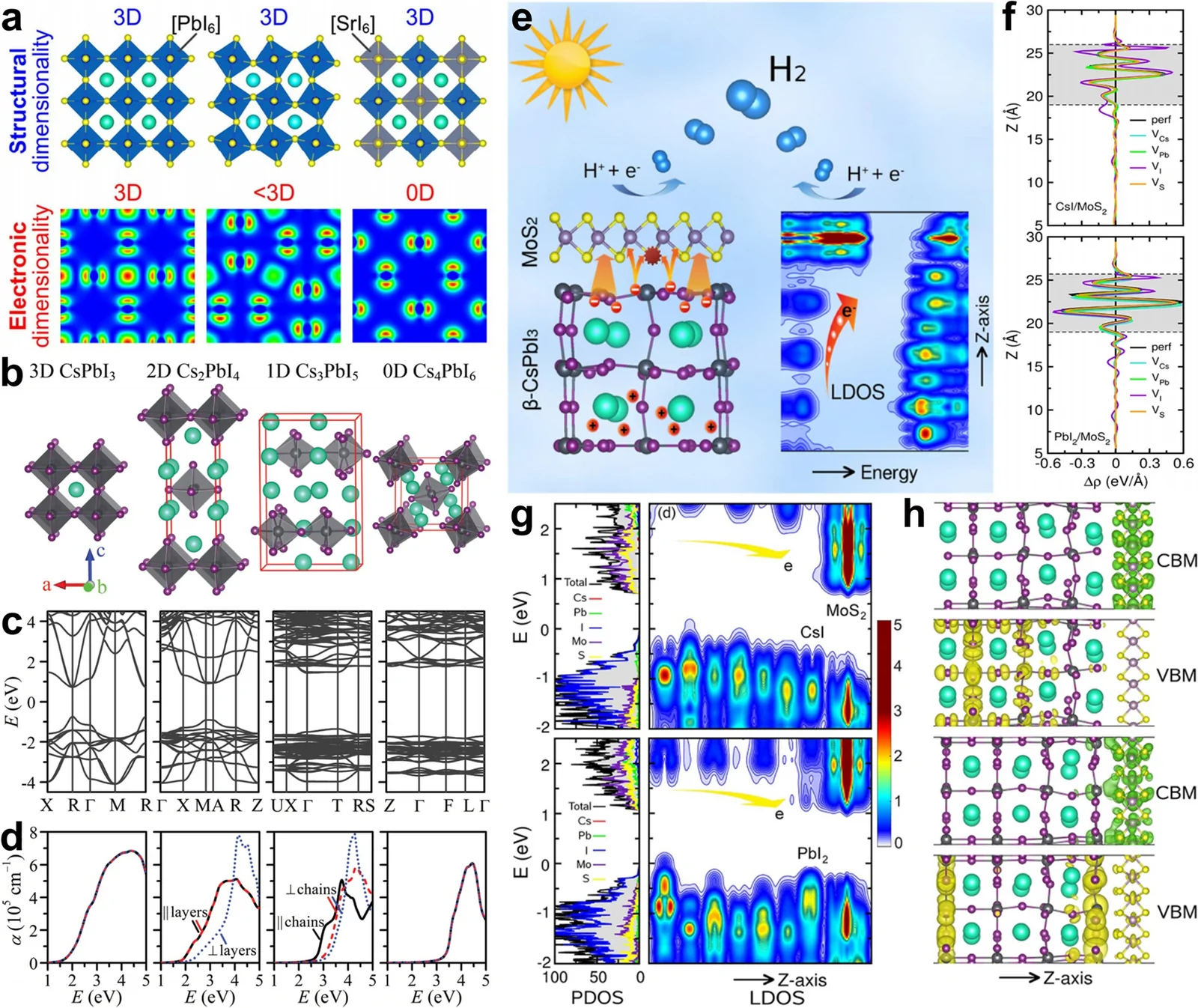
**a** Visual comparison and display of structural dimensionality (above) and electronic dimensionality (below): Taking the perovskite materials as an example. **b** Theoretical crystal structure models of CsPbI_3_, Cs_2_PbI_4_, Cs_3_PbI_5_, and Cs_4_PbI_6_. **c** Computed electronic band structures and **d** simulated optical absorption spectra for the corresponding cesium lead iodide model systems. Reproduced with the permission of Ref. [81] Copyright 2017, Royal Society of Chemistry. **e** PHE mechanism, charge migration behavior, and vacancy feature of CsPbI_3_/MoS_2_ heterostructure. **f** Interfacial charge redistribution: Laterally averaged electron density difference across the interfacial axis. **g** Atomic-projected DOS and *z*-axis projected local DOS isolines; **h** VBM/CBM charge density isosurfaces for CsI/MoS_2_ (top) and PbI_2_/MoS_3_ (bottom) interfaces. Reproduced with the permission of Ref. [170] Copyright 2022, American Chemical Society

In designing high-performance semiconductors for enhanced STE performance, maximizing electronic dimensionality is paramount to achieve intrinsic optoelectronic superiority. Semiconductors exhibiting high structural but low electronic dimensionality suffer from compromised charge transport (barriers to isotropic current flow) and accelerated carrier recombination [164–169], which cannot be resolved via structural optimization alone (Fig. 6a). The electronic dimensionality framework explains why certain systems exhibit anomalous bandgaps or poor STE efficiencies despite favorable structural metrics: Isolated coordination units (e.g., face-sharing bioctahedra) or cation-induced orbital decoupling impose 0D electronic character, even within nominally 3D structures. To realize efficient STE function, semiconductors should exhibit 3D electronic connectivity, ensuring delocalized band edges, shallow defect levels, and minimized carrier recombination [81]. This strategy requires selecting elements and lattice configurations that promote orbital hybridization continuity across all dimensions, transcending structural classifications to unlock optimal light harvesting and charge extraction in advanced STE materials.

Based on the excellent semiconductor properties corresponding to the 3D electronic dimensionality, Xiao et al [81] selected the perovskite CsPbI_3_ to elucidate the importance of 3D electronic dimensionality. They systematically compared the electronic and optical properties of cesium lead iodide compounds with progressively reduced structural dimensionality (3D CsPbI_3_, 2D Cs_2_PbI_4_, 1D Cs_3_PbI_5_, and 0D Cs_4_PbI_6_) and revealed how this directly translates to their respective electronic dimensionality (Fig. 6b–d). Importantly, only the structurally 3D CsPbI_3_ perovskite exhibits a true 3D electronic dimensionality, characterized by highly dispersive CBM (primarily Pb 6*p* states) and VBM (Pb 6*s*–I 5*p* antibonding states) bands along all crystallographic directions. Consequently, CsPbI_3_ demonstrates low, isotropic carrier effective masses, high carrier mobility in all directions, and strong, isotropic optical absorption due to the *p*-*p* transition at its 1.48 eV bandgap. By contrast, the cesium lead iodide compounds with lower structural dimensionality inherently possess lower electronic dimensionality due to restricted orbital connectivity. Specifically, the 2D Cs_2_PbI_4_ (bandgap 1.90 eV) exhibits pronounced band dispersion within the octahedral layers but negligible dispersion perpendicular thereto, leading to carrier confinement within 2D planes and highly anisotropic optical absorption. Similarly, 1D Cs_3_PbI_5_ (indirect bandgap 2.80 eV) exhibits dispersion only along the octahedral chains, with poor cross-chain transport. In the case of 0D Cs_4_PbI_6_ (bandgap 3.44 eV), fully isolated [PbI_6_] octahedra, displays completely flat (non-dispersive) bands in all directions, confirming its 0D electronic dimensionality. This fundamental lack of orbital connectivity results in large effective masses, poor carrier mobility, and a significantly widened bandgap. Therefore, the comparative analysis across 3D CsPbI_3_, 2D Cs_2_PbI_4_, 1D Cs_3_PbI_5_, and 0D Cs_4_PbI_6_ conclusively demonstrates that electronic dimensionality, defined by the spatial connectivity of the atomic orbitals forming the band edges, which is intrinsically linked to structural dimensionality in these model systems and is the primary factor governing critical STE properties like bandgap size, carrier mobility, and optical absorption anisotropy. Accordingly, the 3D electronic dimensionality CsPbI_3_-based materials have been recently applicated in efficient PHE [170–173]. Especially, Ri et al. engineered a CsPbI_3_/MoS_2_ heterostructure photocatalyst, achieving enhanced PHE performance through rigorous first-principles analysis of interfacial defect dynamics [170]. The authors reveal that interfacial iodine vacancies in CsPbI_3_/MoS_2_ heterostructures enhance charge separation by amplifying interface dipole moments (Fig. 6e, f), while sulfur vacancies introduce mid-gap traps (Fig. 6g). Type-II band alignment facilitates electron transfer to MoS_2_ (Fig. 6h), leveraging the high electronic dimensionality of CsPbI_3_ for directional carrier migration. Thus, systematic interrogation of CsPbI_3_’s 3D electronic dimensionality, coupled with its exemplary performance in PHE, provides dual theoretical and experimental guidance for advanced design concept of MS photocatalyst, offering profound implications for next-generation photocatalyst engineering.

On a deeper level, the excellent performance endowed by 3D electronic dimensionality originates from several intertwined microscopic mechanisms that collectively optimize the key steps in PHE at the atomic and orbital levels. Fundamentally, the extension of the orbital hybridization along all crystallographic directions can create spatially delocalized band edges, which directly reduces the effective masses of both photogenerated electrons and holes. This isotropic band dispersion enables rapid and directionally uniformed charge migration, thus minimizing the migration time of photogenerated carriers to surface reaction sites and suppressing bulk/surface recombination. Furthermore, the continuous orbital network inherent to 3D electronic connectivity also favors the formation of shallow defect states, as opposed to deep-level traps (non‑radiative recombination centers). Such excellent defect properties are crucial for maintaining high carrier concentrations and longevity during the PHE process. From the dynamic perspective, the highly dispersive VB and CB effectively enhance the optical transition matrix elements, leading to strong and broad light absorption across the visible-light spectrum. Meanwhile, the delocalized charge density at band edges also effectively facilitates efficient interfacial charge transfer by lowering the energy barrier for carrier injection into the reactive sites. Essentially, the 3D electronic dimensionality orchestrates a synergistic interplay among enhanced light harvesting, accelerated bulk charge migration, inhibited recombination, and favorable surface reaction kinetics. These essential factors jointly constitute the fundamental basis for the dramatic improvements in PHE performance observed in MS photocatalysts.

### Quantitative and Semi-Quantitative Parameters for Determining Electronic Dimensionality

**Fig. 7:**
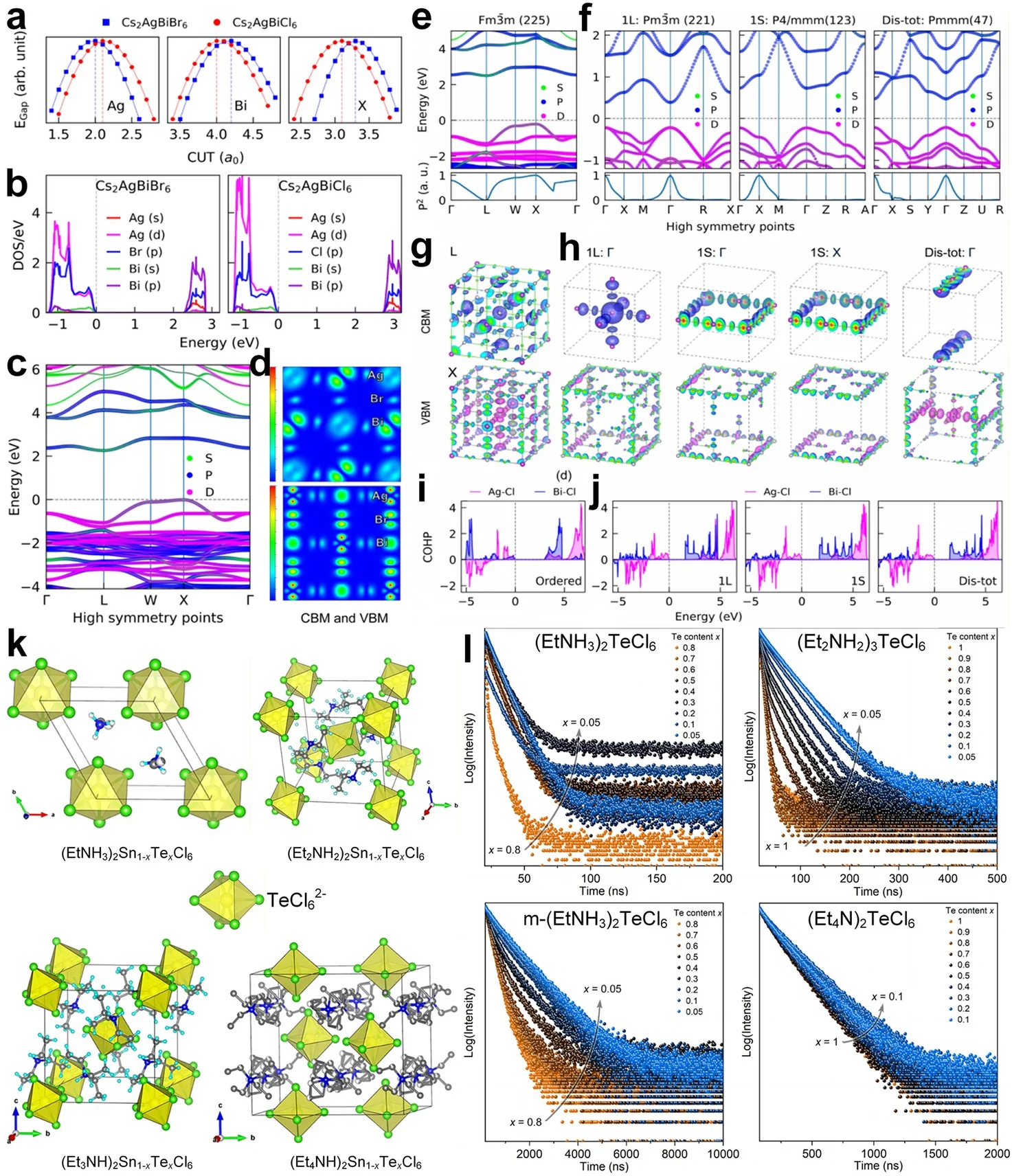
Computational determination and characterization of electronic dimensionality in the halide double perovskite models, illustrating the key parameters and analyses used to assess the electronic dimensionality of Cs_2_AgBiX_6_ (X = Br, Cl) systems, and comparing ordered and cation-disordered structures: **a** Determination of the optimal cut-off parameter for self-energy potential corrections in the DFT-1/2 method. **b** DOS and **c** orbital-projected band structure for ordered Cs_2_AgBiBr_6_, calculated with DFT-1/2 method (The VBM is set to zero energy). **d** Iso-surface plots of the orbital densities for the CBM (top) and VBM (bottom) in ordered Cs_2_AgBiBr_6_, viewed along (100) direction of the cubic unit cell, revealing the spatial confinement of charge carriers. Orbital-projected electronic band structures calculated using the DFT-1/2 method for **e** the ordered phase and **f** representative cation-disordered configurations (1L, 1S, Dis-tot) (The *E*_F_ is set to 0 eV). **g, h** Corresponding orbital density isosurfaces for the CBM (top) and VBM (bottom) at high-symmetry points, illustrating the spatial distribution of the band-edge states. ICOHP analyses for Ag–Cl and Bi-Cl interactions that dominate the band edges in **i** ordered and **j** disordered systems. Positive ICOHP values denote the antibonding character, whereas negative values indicate the bonding interactions (The VBM is aligned at 0 eV). These plots collectively provide a multi-parameter assessment, including the band dispersion, orbital delocalization, and bond-resolved interaction strengths, to quantify the evolution of electronic dimensionality from a low-dimensional confined state in the ordered material toward enhanced 3D connectivity in disordered variants. Reproduced with the permission of Ref. [176] Copyright 2024, Royal Society of Chemistry. Illustrative overview methods for the determination of the electronic dimensionality in semiconductor materials: **k** Crystal structures of hybrid tellurium halides (Et_*n*_NH_4−*n*_)_2_TeCl_6_ (*n* = 1–4), showing isolated TeCl_6_^2−^ octahedra (yellow) surrounded by progressively larger organic cations (C: gray, N: blue, H: light blue, Cl: green). The increasing cation size reduces the inter-octahedral orbital overlap, effectively lowering electronic dimensionality. **l** Corresponding TRPL decay profiles of solid solution systems (Et_*n*_NH_4−*n*_)_2_Sn_1−*x*_Te_*x*_Cl_6_ under 372 nm excitation. The PL lifetime and quenching behavior vary systematically with organic cation size and Te content, reflecting changes in the electronic coupling between neighboring Te centers. Shorter lifetimes and stronger concentration quenching in systems with smaller cations (EtNH_3_^+^) indicate higher electronic dimensionality and enhanced inter-site energy transfer, whereas larger cations (Et_4_N^+^) lead to the longer lifetimes and weaker quenching, consistent with the behavior of electronically isolated 0D. Together, these structural and photophysical analyses provide multi-faceted basis for quantifying electronic dimensionality, linking the geometric arrangement, orbital overlap, and optoelectronic response. Reproduced with the permission of Ref. [175] Copyright 2024, American Chemical Society

While the conceptual framework of electronic dimensionality is powerful for rationalizing the properties of semiconductor material, its actual application greatly relies on clear methodologies for its determination and characterization. Unlike the structural dimensionality directly accessible via diffraction techniques, electronic dimensionality is an emergent quantum property inferred from the accurate electronic structure analysis. Its assessment depends on the multi-faceted suite of computational and experimental descriptors that move beyond qualitative inspection toward the quantitative or semi-quantitative evaluation (Fig. 7) [174–176].

The most fundamental evidence originates from the first-principles calculations of the electronic structure. The spatial delocalization of charge density associated with the VBM and CBM serves as the primary visual indicator: The true 3D electronic connectivity exhibits as continuous, 3D networks of charge density throughout the lattice, whereas lower dimensions exhibit confinement to the planes, chains, and isolated clusters. More quantitatively, the dispersion of key bands, particularly those forming the band edges, is also a critical metric. For instance, in the halide systems, the bandwidth of the σ* CB has been directly correlated with electronic dimensionality, where broader bandwidths signify higher dimensionality and greater orbital overlap [174]. At the same time, this also translates into the effective masses of the carriers. Materials with 3D electronic dimensionality are characterized by low, isotropic effective masses for both electrons and holes, as derived from the band curvature. Significantly anisotropic or high effective masses are the negative factors for reducing the dimensionality, indicating the constrained carrier motion [176]. Moreover, these calculated and computational parameters have direct experimental correlates. The anisotropy in optical absorption spectra, calculated or measured with the polarized light, directly reflects the underlying electronic anisotropy. As a result, the semiconductor material with 3D electronic connectivity exhibits relatively isotropic absorption above the bandgap, while lower dimensionality leads to the strong polarization dependence, typically strong in-plane versus weak out-of-plane absorption [81]. Furthermore, the photophysical properties of emissive centers can act as the sensitive probes of inter-site coupling and, by extension, electronic dimensionality. On the basis of nominally isolated structural units, the degree of the photoluminescence (PL) concentration quenching, carriers’ lifetime, and quantum yield greatly depend on the electronic coupling between these units. The weak coupling (low electronic dimensionality) minimizes concentration quenching, while enhanced coupling (3D electronic dimensionality) facilitates energy transfer and quenching and thus provides an indirect spectroscopic measure of orbital overlap [175].

Beyond accurate density functional theory (DFT) calculations, the innovative crystal structure-based analysis tools also offer rapid, semi-quantitative assessment. Representatively, the Voronoi polyhedron (VP) method, which separates space based on the atomic distances, can quantify non-covalent interactions between the neighboring coordination units. Specifically, by analyzing the faces shared between the VPs of atoms on the neighbor structural units, one can map the strength and dimensionality of the orbital overlap pathways. Significant shared-face areas indicate strong inter-unit coupling and higher electronic dimensionality, even in 0D structural materials, whereas isolated VPs confirm the true electronic isolation [175]. Therefore, this geometric approach also provides a valuable pre-screening tool independent of demanding the electronic calculations. At the same time, the emerging concept of fractional or non-integer electronic dimensionality is also crucial for understanding the complex systems, typically disordered alloys or imperfect networks. In this case, the electronic connectivity may not be uniformly 3D, 2D, or 1D but exist in a middle state. A comprehensive evaluation demands integrating qualitative orbital connection maps along with quantitative measures of covalent bond strength, such as the integrated crystal orbital Hamilton population (ICOHP) integrated up to the Fermi level (*E*_F_). The distribution and strength of the key bonds, typically the metal-anion, along different crystallographic directions dictate the degree of carrier delocalization and anisotropy in each direction, leading to the map with minor difference that governs properties like directional effective masses and absorption [176].

Therefore, determining the electronic dimensionality of a semiconductor material is not reliant on a single parameter but on a convergent analysis of the multiple indicators: calculated band dispersion and charge density topology → derived effective masses and optical anisotropy → experimentally accessible photophysical responses → insightful geometric analysis of the crystal structure. This multi-faceted characterization framework effectively transforms electronic dimensionality from a qualitative concept into a strong and feasible guideline for predicting and engineering effective charge migration and optoelectronic functionality in advanced MS semiconductors.

### Advanced MS Photocatalyst with 3D Electronic Dimensionality

**Fig. 8:**
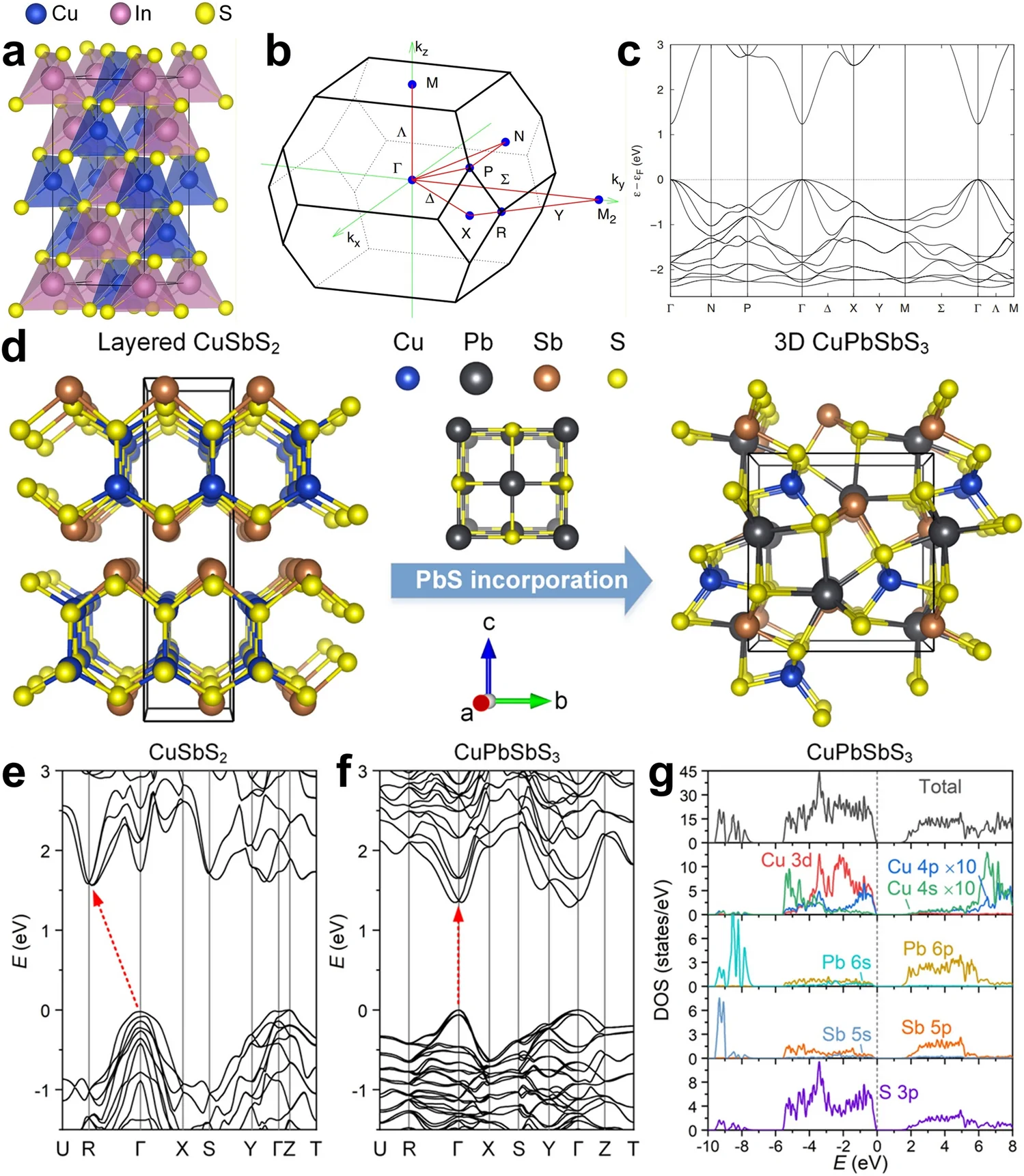
**a** Crystal structure of CuInS_2_ with typical tetragonal chalcopyrite phase. Reproduced with the permission of Ref. [87] Copyright 2023, Elsevier. **b** Brillouin zone, and **c** theoretical calculated band structure of CuInS_2_. Reproduced with the permission of Ref. [177] Copyright 2015, American Physical Society. **d** Structural evolution from layered CuSbS_2_ (left part) to 3D CuPbSbS_3_ (right part) via PbS intercalation (middle part). **e** HSE-calculated band dispersion of CuSbS_2_. **f** SOC-HSE band structure of CuPbSbS_3_. **g** Total/projected DOS for CuPbSbS_3_ under HSE + SOC formalism (Cu 4*s*/4*p* orbitals amplified 10×). Reproduced with the permission of Ref. [194] Copyright 2020, Elsevier

As detailed in Sect. 3, the CuSbS_2_ MMS photocatalyst represents a methodological advancement in MS photocatalysts for PHE, emanating from strategic cationic substitution of Sb for In within the CuInS_2_ lattice (Fig. 8a–c and left part in Fig. 8d) [138, 177]. Notably, lead halide perovskites such as CH_3_NH_3_PbI_3_ demonstrate that Pb^2+^ with its 6*s*^2^ lone pair enables the stabilization of 3D crystal frameworks, giving rise to highly connected electronic structures. This electronic characteristic favors the formation of rocksalt-type structures with octahedral coordination [178–186]. Consistent with this trend, the incorporation of PbS into the layered compound CuSbS_2_ induces a structural reconstruction, yielding the formation of CuPbSbS_3_ which adopts a 3D crystalline architecture. This unique attribute facilitates structural reconstruction of layered MS into ternary 3D frameworks through PbS incorporation [187–190]. Exemplifying this paradigm, integration of PbS into the layer CuSbS_2_ lattice yields 3D bournonite CuPbSbS_3_ (Fig. 8d), as validated across multiple experimental studies [191–193].

Building upon this foundation, our group systematically interrogate the electronic dimensionality of CuPbSbS_3_ through DFT calculations [194], a conceptual framework directly extending the 3D electronic dimensionality paradigm established for CsPbI_3_. Computational analyses unequivocally demonstrate that the CuPbSbS_3_ exhibits 3D electronic dimensionality alongside its structural framework, surpassing the optoelectronic limitations inherent to layered CuSbS_2_. As shown in Fig. 8e, the latter manifests an indirect Heyd–Scuseria–Ernzerhof-calculated (HSE-calculated) bandgap of 1.56 eV, aligning with experimental reports (approximately 1.5 eV) [195–198]. This characteristic, prevalent in lone-pair cation-based layered chalcogenides, impedes efficient carrier transport due to phonon-assisted recombination [199], fundamentally constraining the efficiency of PHE. In stark contrast, the CuPbSbS_3_ achieves a direct band transition at the *Γ* point (HSE: 1.61 eV), with VB/CB extrema exhibiting synergistic orbital hybridization. The observed bandgap overestimation relative to experimental values (1.20 − 1.31 eV) stems from methodological exclusion of spin–orbit coupling (SOC), a critical perturbation for heavy post-transition elements. SOC effects intrinsically reduce bandgaps by lifting orbital degeneracies through *j*-*j* coupling and enhancing VB dispersion via *p*-orbital splitting. Crucially, 3D electronic connectivity in CuPbSbS_3_ enables isotropic carrier mobility (> 150 cm^2^ V^−1^ s^−1^) [194] and attenuates defect-assisted recombination, whereas layered CuSbS_2_ suffers from anisotropic transport (*μ* < 30 cm^2^ V^−1^ s^−1^) [200] and deep-level traps. This electronic dimensionality transition, facilitated by PbS integration, elevates optical absorption coefficients by > 300% across visible spectra while reducing exciton binding energies to < 20 meV, parameters essential for high-efficiency STE conversion. Incorporating SOC reduces the bandgap of CuPbSbS_3_ to 1.29 eV (Fig. 8f, red arrow), primarily due to degeneracy lifting within Pb 6*p* orbitals that lowers the CBM. Furthermore, the *Pmn*2_1_ structure enables significant Rashba splitting under SOC, lifting spin degeneracy and inducing momentum-offset band bifurcation. Consequently, the CB edge experiences minor *Γ*-point displacement, yielding a weakly indirect fundamental gap of 1.29 eV. Notably, the Rashba effect, implicated in prolonged carrier lifetimes within MS photocatalysts [201–203], suggests analogous benefits for CuPbSbS_3_ PHE performance. Projected density of states (DOS) analysis (Fig. 8g) reveals VB/CB extrema comprising hybridized antibonding states: S 3*p* coupled with Cu 3*d*/Pb 6*s*/Sb 5*s* orbitals at VBM, versus Cu 4*s*/Pb 6*p*/Sb 6*p* with S 3*p* orbitals at CBM. This pan-lattice orbital involvement confirms 3D electronic connectivity. Consequently, isotropic band dispersion facilitates efficient ambipolar charge transport.

**Fig. 9:**
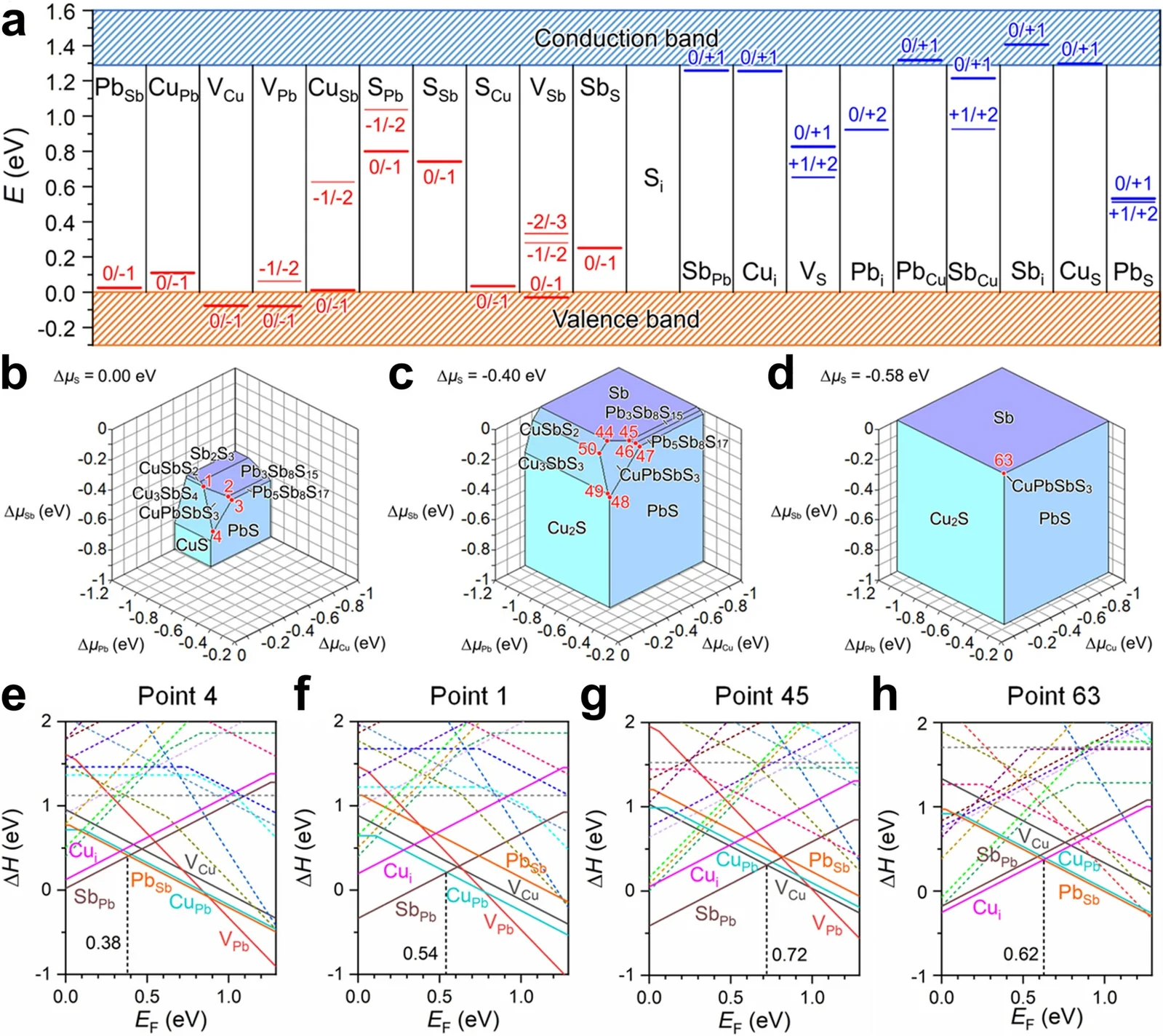
**a** Charge-state transition levels of intrinsic defects in CuPbSbS_3_, derived from DFT calculation. **b–d** 3D visualizations of chemical potentials of Δ*μ*_Cu_, Δ*μ*_Pb_, and Δ*μ*_Sb_ across the Cu–Pb–Sb–S quaternary system, evaluated under discrete Δ*μ*_Sb_ values corresponding to sulfur-rich, sulfur-moderate, and sulfur-poor regimes. **e–h** Formation enthalpies of intrinsic defects in CuPbSbS_3_ as functions of *E*_f_, computed at designated chemical potential points 4, 1, 45, and 63. Reproduced with the permission of Ref. [194] Copyright 2020, Elsevier

The superior defect properties associated with the 3D electronic dimensionality have also been confirmed in CuPbSbS_3_. Quaternary compounds such as kesterite CZTS (mentioned in Sect. 1) exhibit propensity for deep-level cation antisite defects due to isomorphic coordination environments among Cu/Zn/Sn. This disorder is mitigated in Cu_2_BaSnS_4_, in which the steric differentiation between large Ba^+^ occupying [Ba(S/Se)_8_] polyhedra and tetrahedral SnS_4_ units thermodynamically suppresses cation exchange. Analogously, CuPbSbS_3_ features divergent coordination geometries (Fig. 8d) arising from distinct valence states (Cu^+^/Pb^2+^/Sb^3+^), consequently eliminating this defect pathway through inherent chemical incompatibility. Figure 9a shows the charge-state transition levels for intrinsic point defects in CuPbSbS_3_. Six low-formation-energy (Δ*H*_f_ < 1 eV) defects dominate: Cu_i_, Cu_Pb_, Pb_Sb_, Sb_Pb_, V_Pb_, and V_Cu_. Conversely, deep-level defects (V_Sb_, Pb_i_, S_Pb_, and V_S_) exhibit prohibitively high Δ*H*_f_ (> 1 eV), limiting concentrations to 10^6^ cm^−3^ (room temperature) and 10^14^ cm^−3^ (600 K quenched). Crucially, all dominant defects are shallow, V_Pb_, V_Cu_, Cu_Pb_, and Pb_Sb_ acting as acceptors; Cu_i_ and Sb_Pb_ as donors, signifying intrinsic defect tolerance essential for high-efficiency PHE.

The formation enthalpies of intrinsic defects, consequently the equilibrium *E*_f_, exhibit pronounced dependence on synthetic chemical potentials. Figure 9b–d illustrates the ternary chemical space of Δ*μ*_Cu_, Δ*μ*_Pb_, and Δ*μ*_Sb_ under three sulfur regimes: sulfur-rich, sulfur-moderate, and sulfur-poor. Under sulfur-rich conditions, the *E*_f_ resides within the *p*-type region. At chemical coordinate 4 (Fig. 9e), *E*_f_ stabilizes 0.38 eV above the VBM, a regime conferring optimal weak *p*-type conductivity for photovoltaic operation. Progressive sulfur depletion shifts *E*_f_ toward mid-gap (coordinate 1 in Fig. 9f), driven by enhanced formation of *n*-type Sb_Pb_ donors concurrent with suppressed generation of compensating acceptors (Cu_Pb_, V_Cu_, and Pb_Sb_). At intermediate sulfur activity (coordinate 45 in Fig. 9g), *E*_f_ traverses mid-gap by a marginal 0.07 eV, yet the resultant electron concentration (*n* ≈ 10^9^ cm^−3^) falls below Hall-effect detection thresholds. Further reduction to sulfur-poor conditions fails to induce measurable *n*-type conduction; instead, *E*_f_ reverts to 0.62 eV above VBM (Fig. 9h) due to the thermodynamic inhibition of Sb_Pb_ formation. This defect chemistry landscape reveals tunable semiconducting behavior: weak *p*-type (sulfur-rich) transitions to intrinsic character under sulfur-moderate/sulfur-poor conditions. Crucially, sulfur-rich synthesis emerges as the singular pathway to achieve technologically viable hole concentrations (*p* ≈ 10^16^ cm^−3^) while maintaining high hole mobility. Such defect feature, suppressing deep traps while enabling shallow acceptor dominance, constitutes a critical enabler for efficient PHE in CuPbSbS_3_-based photocatalysts.

### General Photocorrosion of MS Photocatalysts

Based on the above discussion, it has been ascertained that the advanced design concept of the 3D electronic dimensionality can effectively guide the design and development of MS photocatalysts with superior PHE performance. Despite significant advances in the rational design of MS photocatalysts through 3D electronic dimensionality (especially CuPbSbS_3_), these materials remain intrinsically susceptible to photocorrosion issue, a critical degradation pathway wherein photogenerated holes oxidize lattice S^2−^ ions [204–208]. Specifically, photocorrosion represents a pervasive challenge that severely compromises the structural integrity and catalytic durability of MS photocatalysts. This issue primarily arises from two interrelated pathways: oxidation by photogenerated holes and reaction with the surrounding medium. Upon light irradiation during PHE, photogenerated holes accumulate on the surface of MS, where they oxidize S^2−^ to elemental sulfur or soluble sulfate species, thereby destroying the material. As a representative instance, in CdS, this process can be described as CdS + 2 h^+^ → Cd^2+^ + S. Concurrently, dissolved oxygen in the aqueous environment acts as an aggressive oxidant, reacting with the photocatalyst to form metal ions and sulfate, further accelerating the destruction. The corresponding key reactions include CdS + O_2_ + 4 h^+^ + 2H_2_O → Cd^2+^ + SO_4_^2−^ + 4H^+^. The synergistic effect of hole-induced oxidation and oxygen-mediated corrosion leads to irreversible structural damage, metal leaching, and significant loss of photocatalytic activity. A profound understanding of these dual mechanisms is essential for designing advanced MS photocatalyst with long-term operational stability. While electronic dimensionality optimization enhances charge separation kinetics and spectral responsiveness, it does not inherently resolve the thermodynamic instability of metal–sulfur (M–S) bonds under photoexcitation. Consequently, even state-of-the-art architectures experience performance decay, undermining their viability for scalable solar fuel production where operational longevity is paramount. Current reliance on sacrificial electron donors (Na_2_S/Na_2_SO_3_, methanol, and triethanolamine) represents a pragmatic yet fundamentally limited approach [201, 209]: Although hole scavenging temporarily stabilizes sulfides, it forfeits energy-storing potential by producing solar energy into chemically inert oxidation products instead of value-added reductants.

This intrinsic trade-off between photostability and PHE efficiency underscores the necessity for innovative stabilization strategies beyond conventional sacrificial chemistry. Merely suppressing photogenerated hole-induced photocorrosion through thermodynamic downhill reactions represents a suboptimal utilization of photonic energy input. To fully harness the benefits conferred by advanced electronic dimensionality control, including enhanced carrier mobility and tailored band alignment, complementary approaches must be developed to either kinetically inhibit sulfur oxidation or thermodynamically redirect hole consumption toward productive reactions. Such methodologies should ideally operate without compromising the redox potential required for target processes like hydrogen evolution. The pursuit of corrosion-mitigating interfaces, kinetic passivation layers, or selective photogenerated hole-transfer pathways thus emerges as an essential frontier in MS photocatalyst engineering. Only by synergistically integrating electronic structure design and corrosion suppression mechanisms can these materials achieve the dual benchmarks of long-term operational robustness and maximal solar energy conversion efficiency demanded by industrial implementation.

## Advanced MS Photocatalysts in PHE Application

Based on the preceding discussion and summarization, the unique 3D electronic dimensionality characteristics of CuPbSbS_3_ have been rigorously established and validated in prior research. Consequently, leveraging this advanced electronic dimensionality design concept, the transition from CuSbS_2_ to CuPbSbS_3_ represents a landmark leap in the development of MS photocatalysts. More recently, our group has implemented the synthesized advanced MS photocatalyst of CuPbSbS_3_ to PHE through a concerted theoretical/experimental approach. Concurrently, addressing the inherent challenge of photocorrosion in MS photocatalysts, we have pioneered the development of corresponding mitigation strategies. In this section, we systematically review the recent advances in the PHE application of state-of-the-art 3D electronic dimensionality CuPbSbS_3_ photocatalyst, alongside effective solutions to the photocorrosion issue in MS photocatalysts. This will serve to precisely delineate the design concept and practical implementation of advanced MS photocatalysts.

### Application of 3D Electronic Dimensionality: The Case of CuPbSbS_3_

The exploration and development of bournonite CuPbSbS_3_ represent a milestone in the rational design of MS photocatalysts with 3D electronic dimensionality. This section systematically outlines the synthesis, structural characteristics, and PHE performance of CuPbSbS_3_, followed by its innovative integration with ferroelectric materials to enable efficient piezo-photocatalytic degradation (piezo-PD), thereby illustrating the versatility of 3D electronic dimensionality in multifunctional photocatalytic systems.

#### Synthesis, Structural Integrity, and PHE Performance

**Fig. 10:**
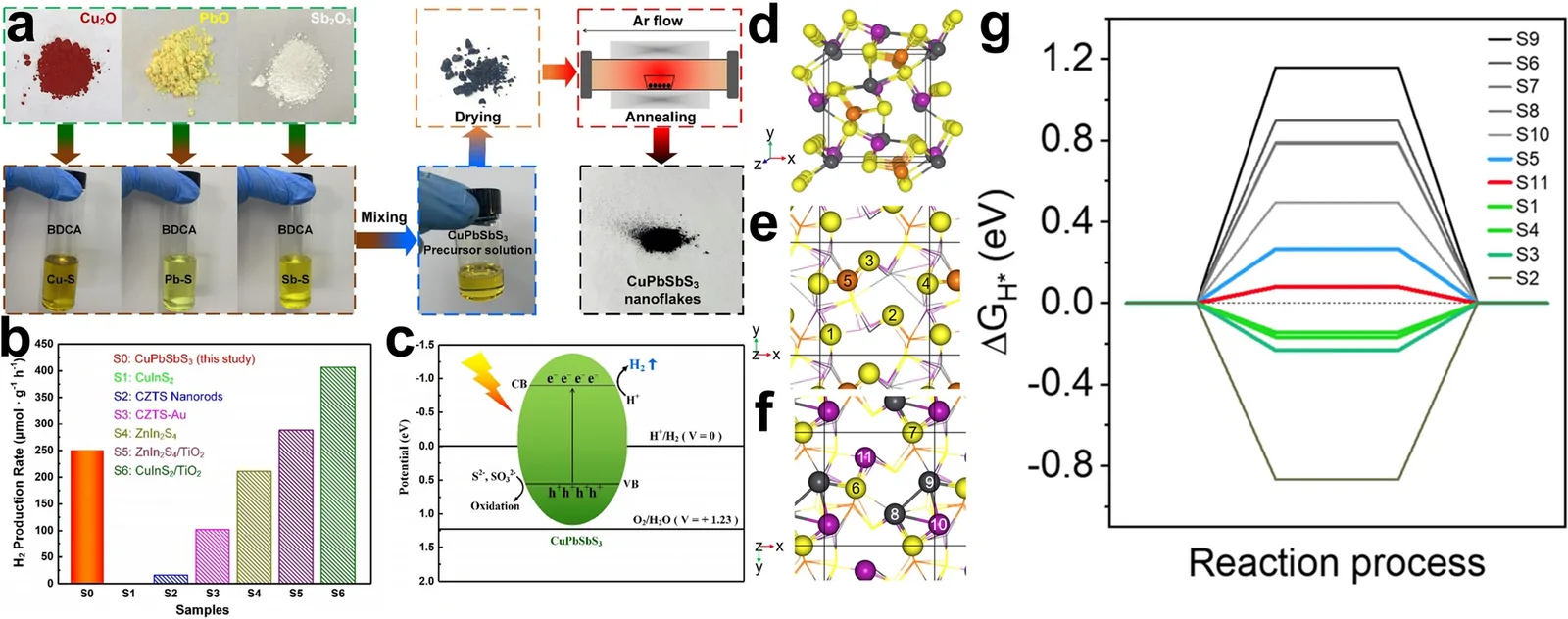
**a** Schematic of the BDCA solution-processing route for bournonite CuPbSbS_3_ nanoflake synthesis. Electron microscopy characterization of BDCA-synthesized CuPbSbS_3_ nanoflakes: **b** Comparative PHE rates under standardized conditions, demonstrating superiority over benchmark sulfides. **c** Proposed mechanistic framework for charge transfer and redox processes. The sequential PHE process involves the steps of (i) electrons’ transfer under light irradiation from the VB (+ 0.54 eV vs. NHE) to the CB (− 0.84 eV vs. NHE), resulting in the formation of photogenerated electron−hole pairs; (ii) efficient separation and migration of photogenerated electrons and holes, facilitated by the 3D electronic dimensionality of CuPbSbS_3_, minimizes the bulk recombination; (iii) photogenerated holes are scavenged by the sacrificial reagents of Na_2_S/Na_2_SO_3_, which concurrently mitigates photocorrosion; (iv) photogenerated electrons in the CB of CuPbSbS_3_, possessing sufficient thermodynamic overpotential, drive the HER via proton reduction. DFT-derived adsorption energetics: **d** crystal structure highlighting corner-sharing octahedra. **e, f** Atomic configuration of (002) facet adsorption sites (1–11); **g** computed hydrogen adsorption Δ*G*_H*_ relative to the thermodynamic optimum (dashed line). Atom color scheme: Cu = orange, S = yellow, Sb = purple, Pb = teal, and H = white. Reproduced with the permission of Ref. [210] Copyright 2022, Elsevier

The experimental CuPbSbS_3_ photocatalyst was successfully synthesized via a butyldithiocarbamate acid (BDCA) solution method (Fig. 10a), yielding phase-pure powders with a predominant (002) crystal orientation [210]. Structural characterization by transmission electron microscopy (TEM) and high-resolution TEM (HRTEM) revealed a stacked-flake morphology and well-defined lattice fringes consistent with the bournonite structure. Energy-dispersive X-ray spectroscopy (EDS) mappings confirmed homogeneous distribution of all constituent elements, underscoring excellent stoichiometric control. Under simulated solar irradiation, the 3D electronic dimensionality CuPbSbS_3_ exhibited a remarkable PHE rate of 250.8 μmol g^−1^ h^−1^ in the absence of cocatalysts, a performance surpassing that of established MS photocatalysts such as CZTS nanorods and ZnIn_2_S_4_, and competitive with state-of-the-art heterojunctions like ZnIn_2_S_4_/TiO_2_ (Fig. 10b). More importantly, the material demonstrated exceptional photostability, with only about 18% decline in activity over 12 h of continuous PHE process, attributable to its robust 3D electronic structure and minimized photocorrosion. The electronic band structure of CuPbSbS_3_, as probed by X-ray photoelectron spectroscopy VB (XPS-VB) and UV–vis spectra, revealed the VB potential of 0.54 eV vs. NHE and a direct bandgap of 1.38 eV, positioning the CB at − 0.84 eV, thermodynamically favorable for proton reduction (Fig. 10c). DFT calculations further elucidated the 3D electronic connectivity within the bournonite framework, characterized by corner-sharing [MX_6_] octahedra (Fig. 10d). The Δ*G*_H*_ calculations on the (002) surface identified several sulfur sites (S1 and S4) with near-ideal Δ*G*_H*_ values (− 0.06 to − 0.08 eV), facilitating the efficient HER kinetics (Fig. 10e–g).

Guided by the electronic dimensionality design paradigm, this study not only establishes CuPbSbS_3_ as a promising photocatalyst for PHE but highlights the critical roles of electronic dimensionality and surface engineering in optimizing catalytic performance. The electronically 3D nature of CuPbSbS_3_ ensures robust charge carrier mobility, while the exposed (002) facet with abundant low-energy catalytic sites enables efficient PHE. These findings pave the way for developing CuPbSbS_3_-based heterojunctions and composite systems, holding great promise for advancing sustainable hydrogen production technologies.

#### ***Integration with BaTiO***_***3***_*** for Enhanced Piezo-Photocatalysis***

Ferroelectric materials are characterized by their non-centrosymmetric crystal structures and the presence of the spontaneous polarization [211–215]. This intrinsic property allows the polarization direction to be switched under the influence of external condition, typically electric field or mechanical stress [216–218]. As a result, these materials offer unique advantages in the electronic and optoelectronic applications. Recently, low-dimensional ferroelectrics have garnered significant research attention due to the synergistic coupling between their piezoelectric/ferroelectric behavior and semiconductor properties, positioning them as promising candidates for semiconductor-based photocatalysis [219]. A representative example is perovskite-type BaTiO_3_, which has been widely investigated owing to its inherent piezoelectric and pyroelectric characteristics [220–222]. Recently, increasing efforts have been devoted to leveraging the cooperative effects between BaTiO_3_ and MS semiconductor photocatalysts [223–227]. Such heterostructures enable the enhancement of optoelectronic carrier dynamics through the modulation of interfacial temperature or the application of mechanical deformation. The spontaneous polarization in ferroelectric materials further induces a built-in electric field, offering a viable strategy for designing advanced photocatalytic systems that harness piezoelectric and thermoelectric optoelectronic effects.

**Fig. 11:**
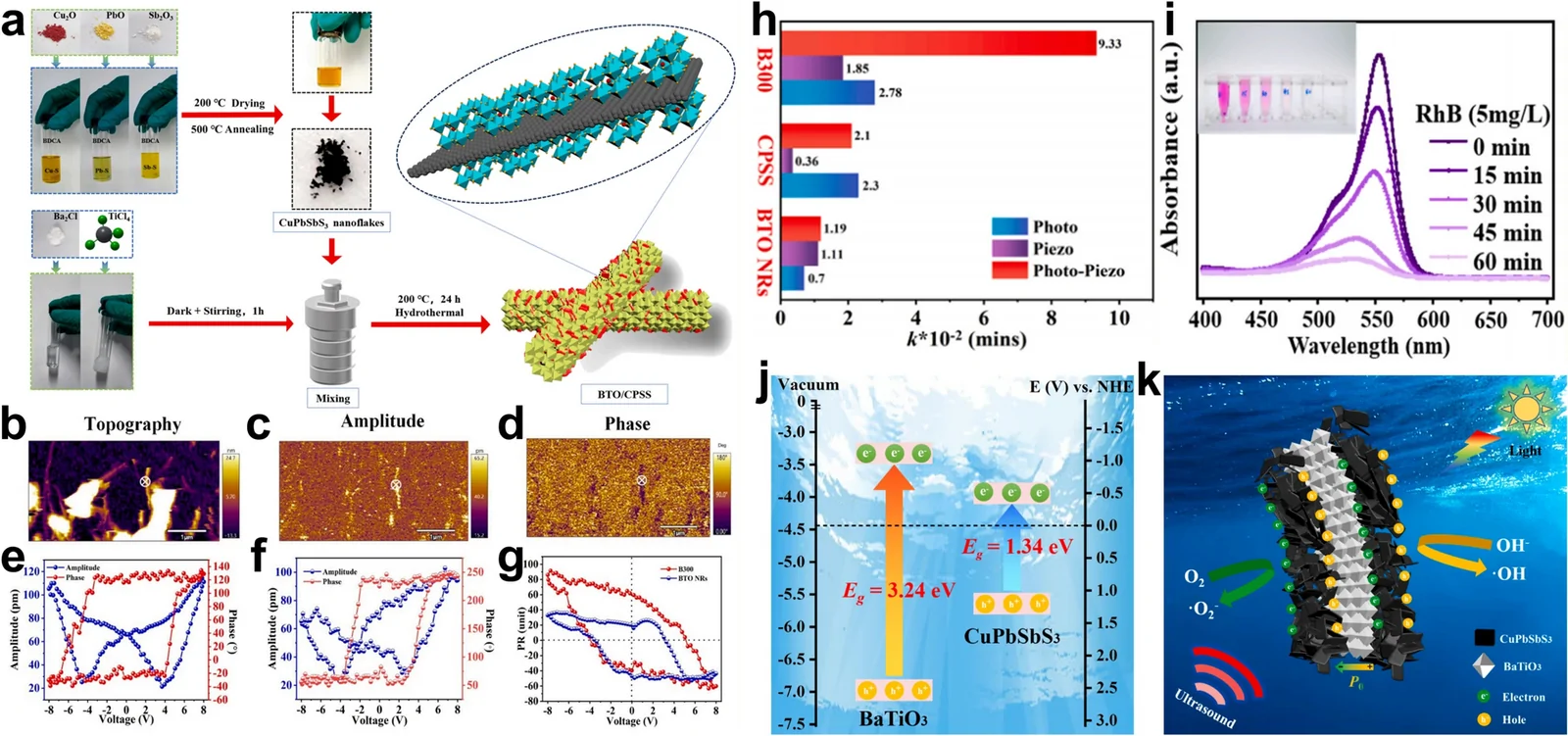
Synthesis, characterization, and enhanced piezo-PD performance of the BaTiO_3_/CuPbSbS_3_ heterostructure. **a** Schematic illustrating the synthesis procedure for the BaTiO_3_/CuPbSbS_3_ heterostructure photocatalyst. Ferroelectric properties were characterized via PFM, presenting: **b** topography, **c** phase, and **d** amplitude images. Corresponding **e** phase hysteresis loops, **f** amplitude loops (“butterfly-shaped”), and **g** calculated piezo-response hysteresis loops are shown for both BaTiO_3_/CuPbSbS_3_ and pristine BaTiO_3_. **h** Comparative analysis of *k* value for RhB degradation across the piezo-PD. **i** Temporal evolution of RhB under piezo-PD conditions, monitored via PL spectra for BaTiO_3_/CuPbSbS_3_. **j** Calculated water redox potentials (E vs. NHE) for the BaTiO_3_/CuPbSbS_3_ heterostructure. **k** Proposed mechanism depicting the enhanced piezo-PD activity within the BaTiO_3_/CuPbSbS_3_ heterostructure. The whole process can be summarized as five steps: (i) Generation of electron−hole pairs in CuPbSbS_3_ by visible-light excitation. (ii) Ultrasonic stress induces piezoelectric polarization in BaTiO_3_ and creates a built-in electric field. (iii) The built-in electric field drives photogenerated electrons to BaTiO_3_ and holes to CuPbSbS_3_ via type-II heterojunction alignment. (iv) Photogenerated holes oxidize adsorbed H_2_O/OH^−^ to •OH, while photogenerated electrons reduce O_2_ to •O_2_^−^. (v) Radicals (h^+^, •OH, •O_2_^−^) collectively mineralize RhB. The synergy effects of BaTiO_3_/CuPbSbS_3_ heterostructure enhances charge separation and suppresses recombination. Reproduced with the permission of Ref. [228] Copyright 2024, Elsevier

The successful exploration of CuPbSbS_3_ based on 3D electronic dimensionality design concept, coupled with its demonstrated efficacy in PHE, has catalyzed significant research interest in its broader photocatalytic applications. Building upon the foundational synthesis of CuPbSbS_3_ via the BDCA solution process and unique features of natural piezoelectric and pyroelectric of BaTiO_3_, Chen et al. further realized the strategic integration of ferroelectric material of BaTiO_3_ through hydrothermal processing [228]. This methodology enabled the construction of hierarchically structured BaTiO_3_/CuPbSbS_3_ heterostructure, wherein the synergistic interplay between BaTiO_3_’s piezoelectric polarization and CuPbSbS_3_’s exceptional optoelectronic properties facilitates efficient piezo-PD of Rhodamine B (RhB). In detail, the BaTiO_3_/CuPbSbS_3_ heterostructures were fabricated via a hydrothermal method using pre-synthesized CuPbSbS_3_ nanoflakes (Fig. 11a). The resulting composite exhibited well-defined interfaces and preserved crystallinity, as confirmed by X-ray diffraction (XRD), scanning electron microscope (SEM), and TEM.

Piezoelectric response force microscopy (PFM) revealed the pronounced ferroelectric behavior in the BaTiO_3_/CuPbSbS_3_ heterostructure, with 180° phase contrast and butterfly-shaped amplitude loops under bias (Fig. 11b-g). The effective piezoelectric coefficient (*d*_33_) of the composite reached 12.1 pm V^−1^, a 135% enhancement over pristine BaTiO_3_, indicating strong polarization coupling at the interface. Under the concurrent ultrasonic and visible-light irradiation, the BaTiO_3_/CuPbSbS_3_ heterostructure achieved a 90.56% degradation of RhB within 30 min, significantly outperforming individual components (Fig. 11h). The optimal loading of 300 mg CuPbSbS_3_ (B300) yielded a first-order rate constant of 9.33 × 10^−2^ min^−1^, underscoring the synergistic piezo-photocatalytic effect. Time-resolved PL (TRPL) spectroscopy corroborated progressive RhB degradation, with near-complete signal quenching after 60 min (Fig. 11i). Meanwhile, the BaTiO_3_/CuPbSbS_3_ heterostructure also demonstrated excellent recyclability, retaining over 80% of its initial activity after multiple cycles. A proposed mechanism (Fig. 11j, k) attributes the enhanced performance to the synergistic interplay between BaTiO_3_’s piezoelectric polarization and CuPbSbS_3_’s semiconductor optoelectronic properties: Visible-light excites electron−hole pairs in CuPbSbS_3_; ultrasonic-induced polarization in BaTiO_3_ creates an internal electric field, facilitating the charge separation; suitable band alignment promotes electron transfer to BaTiO_3_ and hole retention in CuPbSbS_3_; the separated carriers drive radical generation of •OH and •O_2_^−^ and direct oxidation, enabling efficient degradation of RhB.

Building upon the pioneering demonstration of 3D electronically dimensional CuPbSbS_3_ for PHE, this work innovatively integrates CuPbSbS_3_ with ferroelectric material of BaTiO_3_ to construct the BaTiO_3_/CuPbSbS_3_ heterostructure. This synergy achieves exceptional visible-light piezo-PD via enhanced charge separation driven by BaTiO_3_’s polarization field. Therefore, it pioneers CuPbSbS_3_ application in mechano-opto-catalysis and provides a crucial scientific roadmap for designing advanced CuPbSbS_3_-based systems toward more efficient PHE.

### Effective Inhibition of Photocorrosion Issue

**Fig. 12:**
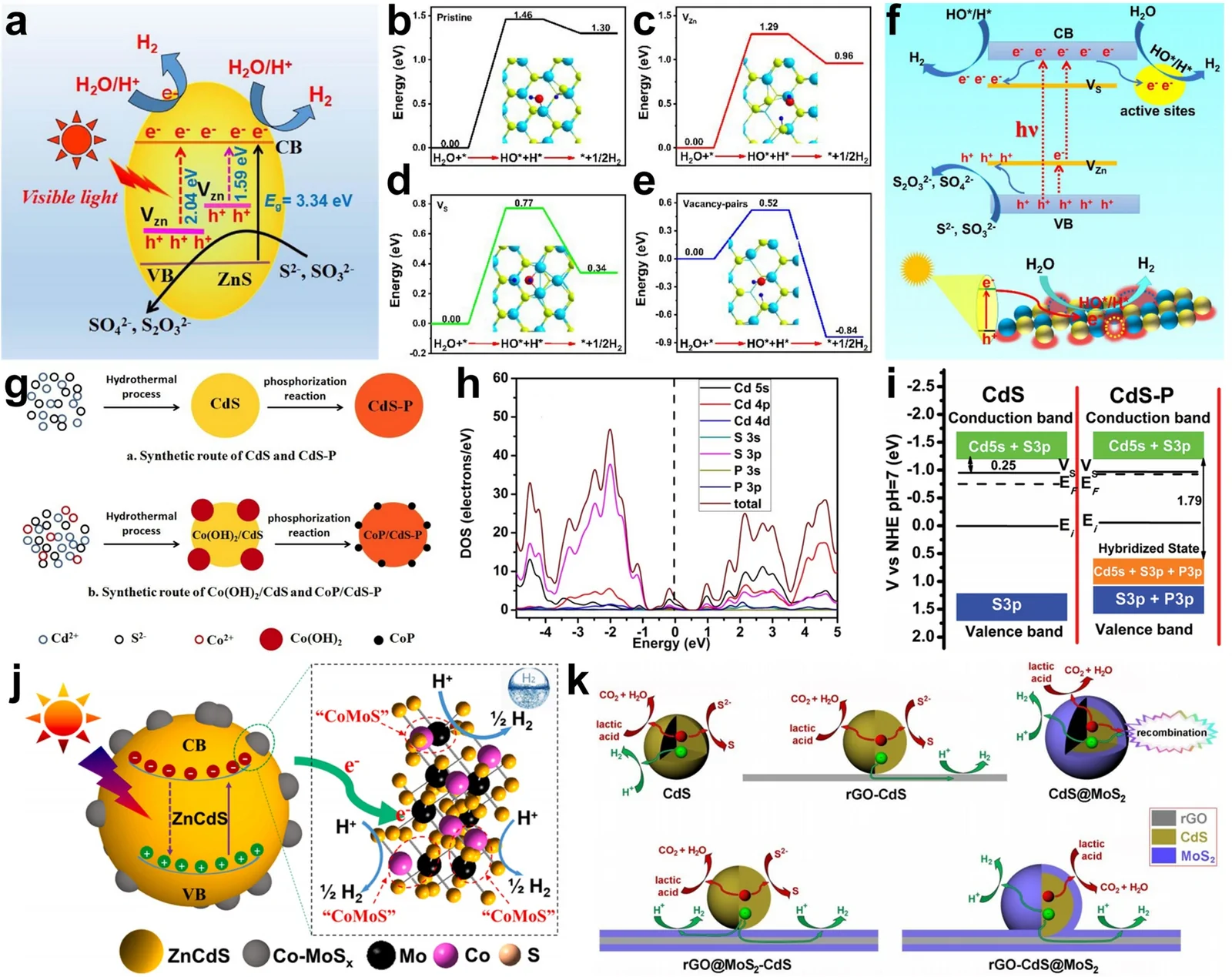
Conventional routes for the solution of MS’s photocorrosion: **a-f** defect engineering; **g-i** elemental doping; **j, k** cocatalyst deposition. **a** Synergistic PHE mechanism of defect-engineered ZnS under visible-light irradiation, illustrating Zn vacancies as multifunctional centers that extend visible-light harvesting, facilitate charge separation, and critically attenuate oxidative photocorrosion through VB modulation. Reproduced with the permission of Ref. [233] Copyright 2018, Elsevier. **b–e** Schematic depiction of the potential energy profile governing PHE, highlighting distinct transition-state geometries on pristine versus vacancy-defective ZnS surfaces. Atomic species are color-coded (Zn: light blue; S: green). **f** Proposed mechanisms for solar-driven photocatalytic hydrogen generation and charge migration over ZnS featuring abundant surface vacancies. Reproduced with the permission of Ref. [234] Copyright 2021, American Chemical Society. **g** Synthesis procedure for Co(OH)_2_/CdS composites (composition tuned via cobalt precursor) and derived CoP/CdS-P, modified from CdS preparation. **h** Room-temperature TRPL decay dynamics of acetone-dispersed nanostructures. **i** Schematic band alignment and *E*_f_ shifts in CdS and CdS-P. Reproduced with the permission of Ref. [235] Copyright 2018, Wiley. **j** Schematic PHE over ZnCdS/CoMoS_*x*_ heterostructures under visible-light illumination. Reproduced with the permission of Ref. [236] Copyright 2021, Elsevier. **k** Schematic PHE over CdS-derived nanostructures with cocatalysts under visible-light. Reproduced with the permission of Ref. [237] Copyright 2021, Royal Society of Chemistry

**Fig. 13:**
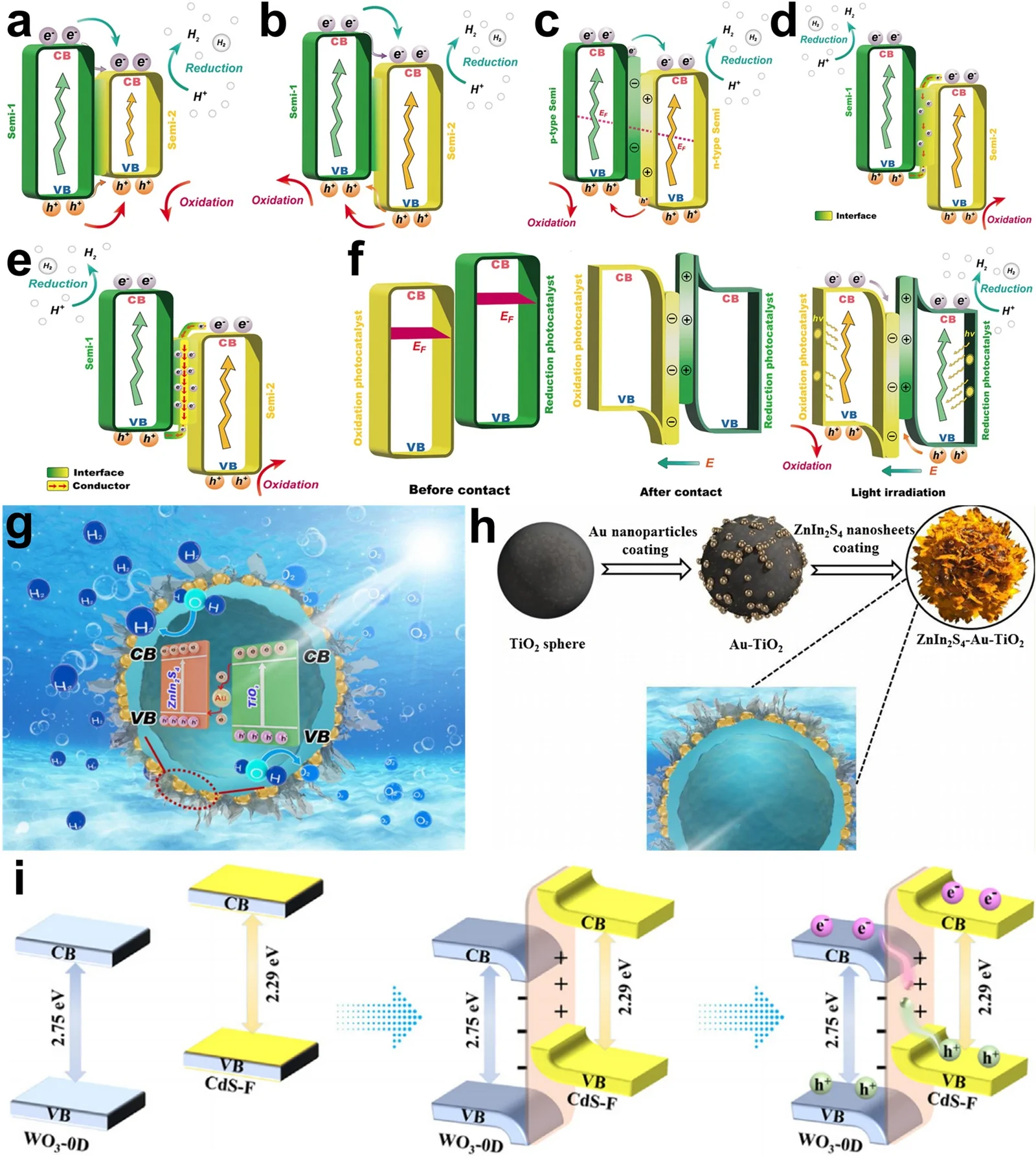
Conventional routes for the solution of MS’s photocorrosion: heterostructure engineering. Charge transfer schematics in heterojunction photocatalysts: **a** type-I, **b** type-II, **c** *p*−*n*, **d** mediator-free Z-scheme, and **e** mediator-assisted Z-scheme. **f** S-scheme interfacial dynamics (pre-contact, post-contact, illuminated states). Reproduced with the permission of Ref. [63] Copyright 2025, Elsevier. **g** Proposed Z-scheme heterojunction in the ZnIn_2_S_4_/Au/TiO_2_ heterostructure for PHE. **h** Two-step fabrication of the photocatalyst: chemical deposition of Au/TiO_2_ followed by solvothermal growth of ZnIn_2_S_4_. Reproduced with the permission of Ref. [238]. Copyright 2018, Elsevier. **i** S-scheme heterojunction mechanism in CdS/WO_3_, showing band alignment and charge separation under illumination. Reproduced with the permission of Ref. [239]. Copyright 2024, Elsevier

As established in Sects. 2.3 and 4.3, the photostability of MS photocatalysts is critically compromised by the photocorrosion issue, a phenomenon driven by surface accumulation of photogenerated holes and extensively documented in prior reviews. Throughout the evolution of MS-based PHE systems, significant research efforts have focused on stabilizing vulnerable lattice S^2–^ ions. Key mitigation strategies encompass defect engineering [229], elemental doping [230], cocatalyst deposition [57, 231], and heterostructure engineering [65, 232], which are also the approaches concurrently employed to enhance overall PHE efficiency of MS photocatalysts (Figs. 12 and 13). Within this context, we first consolidate established methodologies for suppressing photocorrosion in MS photocatalysts and then highlight the emerging paradigm of “controllable-photocorrosion” paradigm for designing ultra-stable MS photocatalysts with precision-engineered degradation pathways.

#### Conventional Routes for the Solution of Photocorrosion

Defect engineering constitutes a strategic modification approach for extending the optical absorption range of MS and reducing the adsorption and activation energies of reactive intermediates [240–247]. Typically, due to their lower formation energy compared to cation vacancies, surface sulfur vacancies are preferentially employed to modulate the local coordination environment, thereby enhancing PHE activity. However, such vacancies are often insufficient for stabilizing lattice S^2−^ ions, primarily attributed to inefficient photogenerated hole extraction dynamics. Conversely, the introduction of cation vacancies can elevate the VB position through the formation of defect levels above the VBM. This elevation effectively diminishes the oxidizing power of photogenerated holes, offering a pathway toward enhanced the stability of MS photocatalyst. This principle has been exemplified in zinc-deficient ZnS systems, where deliberate fabrication via sulfur source control generates Zn vacancies [233]. These vacancies demonstrably attenuate hole oxidation capacity, significantly mitigating the inherent photocorrosion tendency of ZnS. To synergistically harness the benefits of defect engineering for concurrent activity and stability enhancement, the strategic integration of both cation and anion vacancies within MS photocatalysts has emerged as a promising direction. Supporting this concept, contemporary research has successfully engineered surfaces rich in both zinc and sulfur vacancies on ZnS [234]. Within this dual-vacancy configuration, sulfur vacancies primarily facilitate the tailoring of surface electronic properties to activate adsorbed water molecules, whereas zinc vacancies contribute critically to stability by modulating the VB position upward.

Elemental doping, encompassing both cationic and anionic substitutions, represents a pivotal strategy for modulating the energy band structure of MS photocatalyst [248–254]. Specifically, the introduction of foreign atoms can effectively regulate the oxidation potential of photogenerated holes by establishing impurity energy levels within the bandgap or by forming solid solutions, thereby constituting a highly promising approach for enhancing the PHE stability of MS photocatalysts. Illustrative of this principle, gradient phosphorus doping within CdS nanostructures has been demonstrated to induce an oriented built-in electric field [235]. This internal field facilitates the efficient extraction of photogenerated holes from the bulk to the surface. Consequently, a significant suppression of photocorrosion is realized, as sulfur sites located in the interior are effectively shielded, while photogenerated holes are proficiently extracted and consumed. Such architectural design provides a valuable paradigm for engineering stable and high-performance MS photocatalysts to meet scale-up application requirements. As anticipated, the P-doped CdS exhibited substantially enhanced PHE activity, maintaining robust performance over multiple reaction cycles without significant decreasing. Similarly, the incorporation of transition metal cations (like Zn, Co, Mn, Fe etc.) has also proven effective in mitigating the inherent photocorrosion susceptibility of CdS to varying extents [255].

Cocatalyst deposition has been extensively explored as a strategic approach to divert photogenerated holes away from MS, thereby mitigating oxidative degradation of these semiconductor materials [256–266]. Specifically, oxidative cocatalysts fulfill the fundamental role of photogenerated hole extraction. For instance, Lei et al. demonstrated that the photodeposited cobalt-modified amorphous molybdenum sulfide (Co-MoS_*x*_) acts as an effective cocatalyst on ZnCdS, significantly enhancing visible-light PHE (551.48 μmol h^−1^, AQE = 21.7%) [236]. Crucially, the Co-MoS_*x*_ layer synergistically suppresses ZnCdS photocorrosion by facilitating charge separation and forming a stabilized heterointerface, thus addressing a major stability limitation while boosting activity. At the same time, Liu et al. presented a robust strategy to concurrently enhance the PHE activity and photostability of CdS by constructing a stacked nanostructure with reduced graphene oxide (rGO) supporters and metallic MoS_2_ overlayers [237]. Critically, this design effectively mitigates the inherent photocorrosion of CdS, through a dual protective mechanism: the MoS_2_ cocatalyst extracts photogenerated holes, preventing oxidative consumption of S^2−^, while the rGO rapidly shuttles excess electrons to MoS_2_ for efficient proton reduction. This synergistic cocatalyst integration yields exceptional PHE rates (14.4 mmol g^−1^ h^−1^) and remarkable cyclic stability due to the effective inhibition of photocorrosion, underscoring the pivotal role of tailored cocatalyst deposition in overcoming stability challenges in MS-based photocatalysis.

Beyond the aforementioned-approaches, heterojunction engineering represents a well-established and critical methodology meriting particular emphasis [267–273]. To date, primary heterojunction configurations encompass type-I, type-II, *p* − *n*, Z-scheme, and S-scheme architectures [274–280]. Illustratively, the intrinsic photocorrosion susceptibility of ZnIn_2_S_4_ has been successfully addressed through constructing a ternary Z-scheme system comprising TiO_2_, Au, and ZnIn_2_S_4_ [238]. This configuration facilitates interfacial recombination of holes from ZnIn_2_S_4_ with electrons from TiO_2_ via the Au electron mediator. Liu et al. demonstrated that constructing S-scheme heterojunctions by integrating WO_3_ with MS (typical maple leaf-shaped CdS) significantly enhances PHE activity [239]. The optimized 0D WO_3_/CdS S-scheme heterojunction achieves an exceptional PHE rate of 34.12 mmol g^−1^ h^−1^, attributed to synergistic morphology effects and interfacial charge dynamics. Critically, the S-scheme mechanism effectively mitigates photocorrosion in sulfides by directing photogenerated holes from sulfides to WO_3_, thereby preserving structural integrity. Concurrently, efficient electron−hole separation via the S-scheme pathway and exposure of high-activity (100) crystal facets collectively boost hydrogen generation.

Conventional MS photocatalysts modifications partially suppress photocorrosion but still face two major challenges: (i) synthesis complexity involving demanding operational protocols hinders industrial scalability; (ii) predominant reliance on hole extraction mechanisms fails to prevent lattice S^2−^ oxidation by electron/oxygen-derived superoxide radicals, necessitating co-extraction of both charge carriers. Consequently, the exploration of advanced processes to overcome the photocorrosion issue is of great importance for the design concept of advanced MS photocatalysts.

#### Advanced Design Concept of Photocorrosion Inhibition

Existing photocorrosion mitigation strategies for MS photocatalysts (Sect. 5.2.1), are largely post-synthesis modifications that do not tackle the root causes of photocorrosion. Furthermore, conventional synthetic routes overwhelmingly rely on the introduction of extraneous sulfur sources, such as thioacetamide, sodium sulfide, and thiourea, which both hinder corrosion suppression and generate high-density sulfur vacancies. These high-density vacancies significantly diminish the concentration of photogenerated electrons available for the HER, thereby substantially compromising hydrogen production efficiency. Consequently, the development of advanced synthetic methods capable of fundamentally suppressing MS photocorrosion issue while maintaining rigorous phase purity represents a sophisticated and promising design concept.

**Fig. 14:**
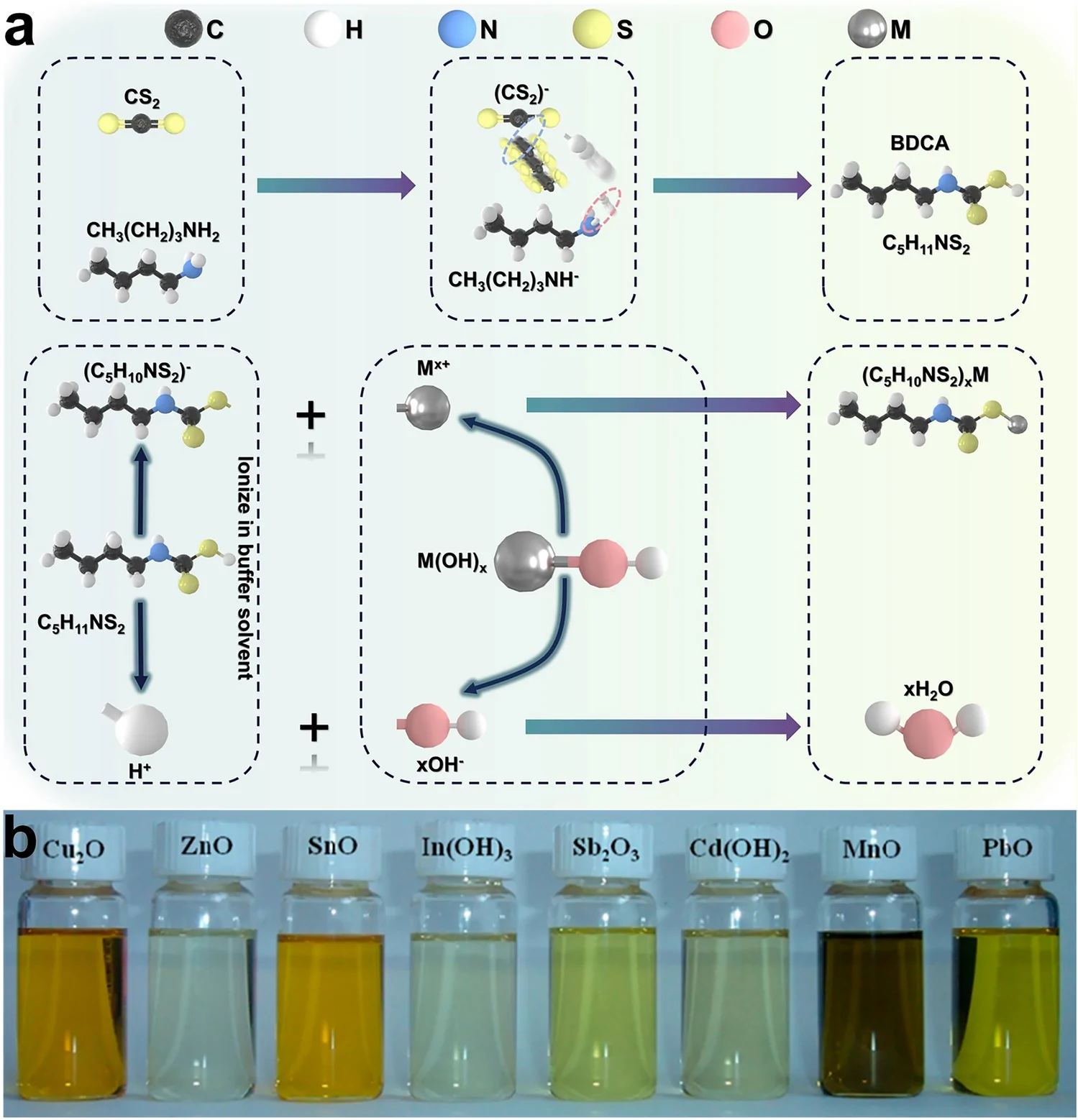
**a** Reaction mechanism for intrinsic sulfur-coordination directionality BDCA solution generation (top) and ambient-temperature dissolution dynamics of MOs/hydroxides in BDCA (bottom). **b** Optical images of BDCA solution after dissolving several representative MOs or metal hydroxides. Reproduced with the permission of Ref. [287] Copyright 2012, American Chemical Society

As detailed in Sect. 5.1.1, CuPbSbS_3_ photocatalysts have been successfully synthesized by the BDCA solution method and applied to PHE. Originally developed depositing absorber layers in thin-film solar cells, typically Sb_2_S_3_, Sb_2_(S, Se)_3_, Cu(In,Ga)(S,Se)_2_, and CZTS thin films’ deposition [281–287], the BDCA solution process lacks targeted mechanistic analysis in the MS context. To address this gap, our group has conducted a focused investigation into the microscopic evolution of the BDCA solution process formation and its corresponding synthesis pathway for MS semiconductor materials (especially the MS photocatalyst) (Fig. 14) [288], revealing its intrinsic “sulfur-coordination-directed” mechanism. This fundamental understanding thereby provides a rigorous scientific foundation for developing advanced synthesis process underpinned by intrinsic sulfur-coordination directionality, offering a promising route toward substantially suppressing MS photocorrosion issue [84]. Specifically, BDCA synthesis proceeds via the reaction of carbon disulfide (CS_2_) with *n*-butylamine (CH_3_(CH_2_)_3_NH_2_) [281]. During this chemical transformation, homolytic cleavage of one C = S bond in CS_2_ generates a reactive S = C–S• radical intermediate. Subsequently, the unsaturated carbon and sulfur atoms within this intermediate undergo concerted reactions with the nitrogen and hydrogen atoms of *n*-butylamine, respectively, culminating in the formation of BDCA (C_5_H_11_NS_2_). Within the C_5_H_11_NS_2_ structure, the dithiocarbamate moiety (−NH−CSS−) exhibits dual functionality, serving concurrently as a sulfur donor and a metal-chelating ligand. This bifunctional nature fosters a dynamic coordination equilibrium involving metal–sulfur interactions at the molecular level, endowing the system with its distinctive intrinsic sulfur-coordination directionality property. Furthermore, the thionothiolic acid group (−CSSH) in BDCA undergoes facile deprotonation in ethanolic solution, yielding H^+^ and S_2_CNHC_4_H_9_^−^ ions. The liberated protons promote the dissolution of MO (or metal hydroxide) precursors, forming an organometallic complex, M(S_2_CNHC_4_H_9_)_*x*_, with water (*x*H_2_O) as a byproduct. Following drying and pyrolysis, the MS photocatalyst is ultimately formed via the thermal decomposition pathway: M(S_2_CNHC_4_H_9_)_*x*_ → M_*y*_S_*x*_ + *x*(S_2_CNHC_4_H_9_). Therefore, based on the deep analysis of the BDCA solution formation mechanism and the microscopic evolution of MS synthesis via the BDCA method, it can be revealed that the −NH−CSS− organic group within BDCA molecular exhibits dual functionality, concurrently serving as both the sulfur source and a metal-chelating ligand. This inherent bifunctionality enables the direct synthesis of MS semiconductor materials (especially MS photocatalysts) without requiring extraneous sulfur sources, thereby significantly streamlining the synthetic protocol while concurrently enhancing phase purity. More significantly, the intrinsic sulfur-coordination directionality property of the BDCA solution facilitates the construction of a molecular-scale metal–sulfur dynamic equilibrium system. This system effectively enhances the M-S bond stability within MS photocatalysts, leading to a substantial suppression of the oxidation reaction between S^2−^ anions and photogenerated holes under light illumination. Consequently, this mechanism provides an effective pathway for mitigating the inherent photocorrosion issue intrinsic to MS photocatalyst. To date, the intrinsic sulfur-coordination directionality property of the BDCA solution method has been effectively validated in the MS photocatalysts of CdS and ZnCdS, demonstrating its substantial efficacy in suppressing the photocorrosion issue. Notably, the deep research on ZnCdS photocatalysts synthesized via this approach has further enabled the proposal of an advanced “controllable-photocorrosion” concept. This paradigm shift strategically transforms the inherent detrimental effects of photocorrosion into a functionally advantageous process [84].

**Fig. 15:**
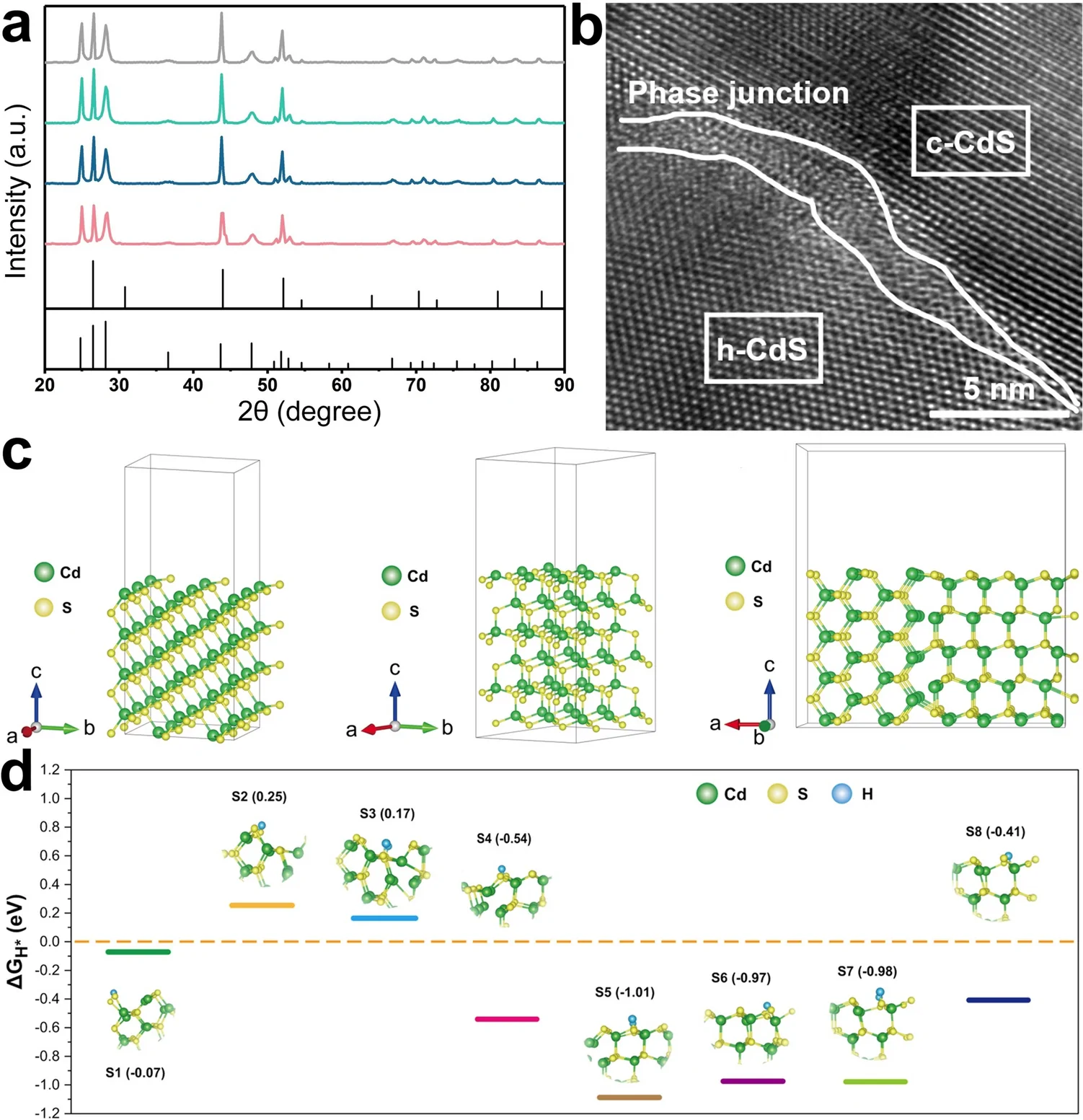
Characterization, and DFT-calculated theoretical insights of BDCA-synthesized CdS photocatalysts: **a** XRD patterns confirming the crystalline phases of CdS samples synthesized following thermal treatment at various temperatures. **b** HRTEM image providing direct evidence of the h-CdS/c-CdS phase junction microstructure. **c** Computational models depicting the atomic configurations of cubic CdS (left), hexagonal CdS (middle), and the constructed phase junction interface (right). **d** Comprehensive mapping of the calculated Δ*G*_H*_ across all sulfur sites identified at the CdS phase junction interface. Reproduced with the permission of Ref. [299] Copyright 2023, Wiley

CdS, an archetypal BMS photocatalyst, possesses an optimal bandgap (~ 2.4 eV) and sufficiently negative CB potential, rendering it highly effective for visible-light-driven PHE [289]. To date, CdS-based photocatalytic systems have achieved significant and noteworthy progress in this domain [289–294], and several comprehensive reviews have extensively documented the research trajectory and future prospects for CdS photocatalysts [295–298]. Notably, the vast majority of both primary research and review articles concerning CdS explicitly acknowledge its severe susceptibility to photocorrosion issue. Consequently, the proposed mitigation strategies predominantly align with the conventional approaches to suppressing photocorrosion, as detailed in Sect. 5.2.1. In light of this persistent challenge, our group pioneered the application of the BDCA solution method to CdS photocatalyst synthesis in 2023, concurrently initiating a preliminary investigation into its underlying mechanism for photocorrosion suppression [299]. Specifically, CdS synthesis initiates with the dissolution of Cd(OH)_2_ in BDCA solution (also shown in Fig. 14b), forming the Cd–S precursor complex. The subsequent thermal treatment involves precise drying at low temperature to remove volatile solvents and annealing at high temperature to crystallize CdS. Crucially, the BDCA serves a dual function: the sole sulfur source, eliminating the need for toxic external sulfur precursors; the metal-chelating ligand, facilitating molecular-scale control over Cd–S bond formation. This inherent intrinsic sulfur-coordination directionality property is central to suppressing high-density sulfur vacancy formation, also the key initiator of photocorrosion issue. XRD analysis (Fig. 15a) confirmed the formation of crystalline CdS across annealing temperatures from 300 to 600 °C. While a mixture of cubic (c-CdS) and hexagonal (h-CdS) phases spontaneously formed, the ratio evolved with temperature: Higher annealing temperatures favored the hexagonal phase. At the same time, the absence of impurity peaks underscores the high phase-purity achievable with BDCA, a direct consequence of its integrated sulfur supply, and chelation capability, mitigating defect-induced instability. The HRTEM provided direct evidence of interfacial regions between c-CdS and h-CdS domains within individual nanoparticles (Fig. 15b). While this phase junction (incidentally formed during synthesis) contributes to charge separation, the primary focus here is the structural integrity. The clean interface and well-defined lattices observed suggest minimal crystallographic defects, attributable to the controlled precursor decomposition of BDCA solution method. This defect suppression is intrinsically linked to enhanced photocorrosion resistance.

Photocatalytic evaluation revealed a strong dependence of PHE rate on annealing temperature. The CdS-500 sample (annealed at 500 °C) achieved a remarkable PHE rate of 7.294 mmol g^−1^ h^−1^ under visible-light without loading of any cocatalysts, surpassing many reported CdS-based systems [296]. Significantly, over four consecutive 4-h cycles (960 min total irradiation), the CdS photocatalyst maintained robust activity with negligible decay and even exhibited a rate increase in the third cycle, indicative of surface activation rather than degradation. This sustained performance starkly contrasts the rapid deactivation typical of bare CdS and directly validates the efficacy of BDCA solution method in mitigating photocorrosion issue of MS, along with other kinds of MS photocatalysts. The inherent sulfur richness and optimized Cd–S bonding environment provided by the BDCA-derived precursor minimize the oxidation of lattice S^2−^ by photogenerated holes, the core mechanism of photocorrosion issue mentioned in Sect. 4.3. DFT models of c-CdS, h-CdS, and their phase junction (Fig. 15c) were analyzed for ΔG_H*_ on sulfur sites (Fig. 15d), revealing that the sulfur sites in pure c-CdS (Δ*G*_H*_ =  + 1.22 to 2.30 eV) and h-CdS (ΔG_H*_ = − 1.35 to − 2.44 eV) exhibited overly weak or strong hydrogen binding, respectively, hindering efficient HER. Obviously, specific sulfur sites near the c-CdS/h-CdS interface achieved near-optimal Δ*G*_H*_ (nearly 0 eV). The charge density redistribution at the junction modulates the reactivity of these interfacial sulfur atoms. Crucially, beyond promoting HER kinetics, the BDCA-derived synthesis likely stabilizes these interfacial sulfur sites. The precise sulfur-coordination during precursor formation and decomposition minimizes undercoordinated or vacancy-associated sulfur sites, which are particularly vulnerable to oxidative attack by photogenerated holes. This inherent stabilization, coupled with thermoneutral Δ*G*_H*_, underpins both high activity and suppressed photocorrosion issue. Under illumination, photogenerated electrons in the higher-lying c-CdS CB migrate to h-CdS, while holes move in the opposite direction across the VB. This spatial separation reduces charge recombination. More importantly, the BDCA-synthesized structure promotes efficient hole consumption at the interface or within the h-CdS domain, likely facilitating their reaction with sacrificial agents (S^2−^/SO_3_^2−^) before they can oxidize the vulnerable lattice S^2−^. Furthermore, the robust Cd–S bonding environment, inherent to the BDCA-derived lattice, intrinsically raises the activation barrier for the photocorrosion reaction (2 h^+^ + CdS → Cd^2+^ + S^0^).

**Fig. 16:**
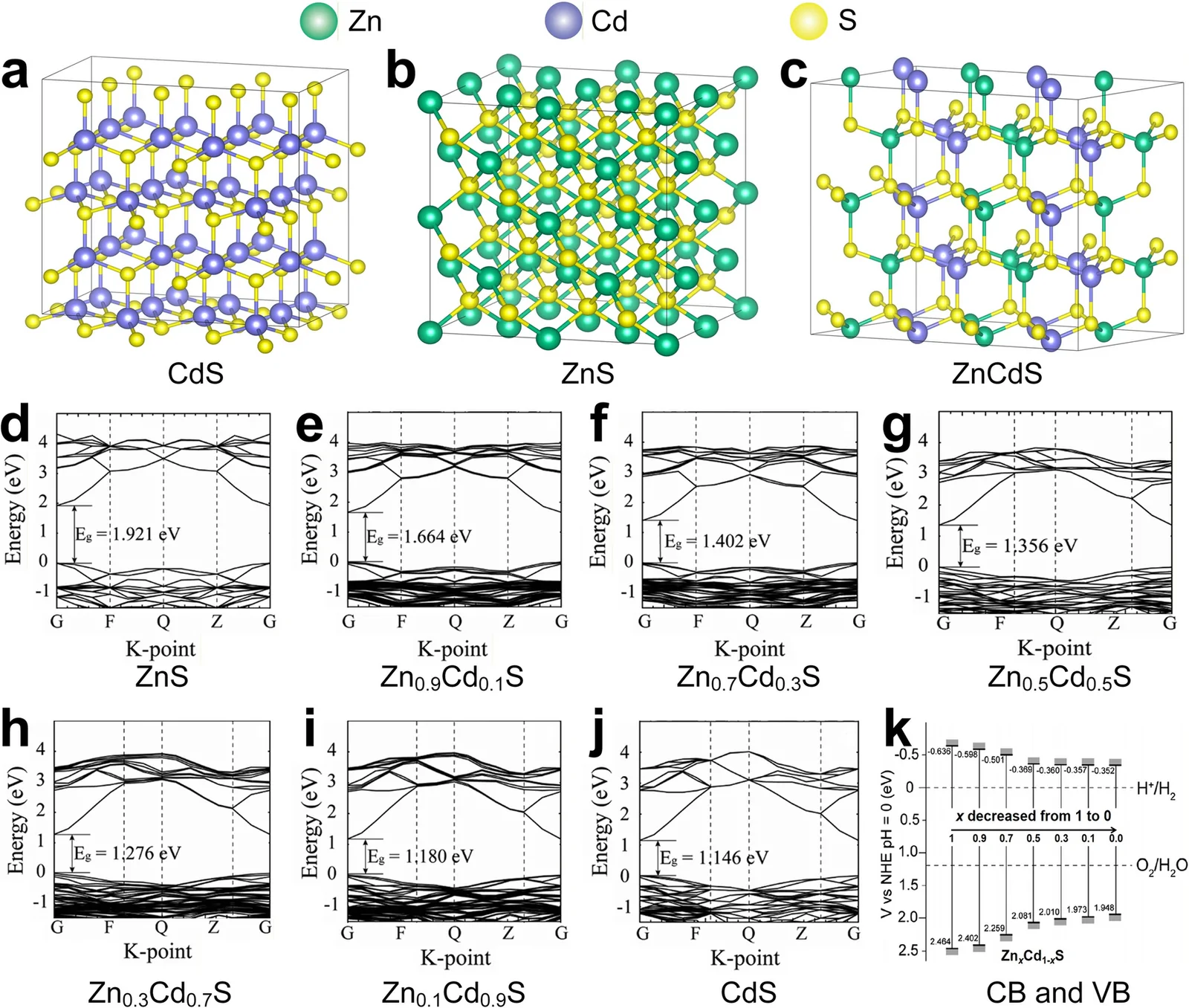
Properties of ZnCdS solid solution, illustrated by crystal structure, DFT-calculated band structure, and CB/VB positions: Crystal structures of **a** CdS, **b** ZnS, and **c** ZnCdS solid solution with Zn/Cd ration of 1:1. Reproduced with the permission of Ref. [65] Copyright 2024, Wiley. DFT-calculated band structures of ZnCdS photocatalysts with different Zn/Cd ratios: **d** 1:0, **e** 9:1, **f** 7:3, **g** 1:1, **h** 3:7, **i** 1:9, and **j** 0:1. **k** CB and VB positions of ZnCdS with different Zn/Cd ratios. Reproduced with the permission of Ref. [301] Copyright 2013, American Chemical Society

Based on the above-mentioned analyses, this research pioneers the intrinsic sulfur-coordination directionality BDCA solution method as a transformative strategy for synthesizing the traditional MS photocatalysts of CdS with intrinsically suppressed the general photocorrosion issue. By utilizing BDCA’s dual role as a sulfur source and chelating ligand, this method ensures the solution of the following four aspects: (i) High phase-purity and reduced defects: Eliminating extrinsic sulfur sources minimizes sulfur vacancies, a primary photocorrosion initiation site. (ii) Optimized Cd–S bonding: The intrinsic sulfur-coordination directionality feature fosters a stable sulfide lattice less susceptible to oxidative hole attack. (iii) Inherent interfacial stability: The naturally formed phase junction, while aiding charge separation, also hosts sulfur sites stabilized by charge redistribution, exhibiting both high HER activity and resistance to oxidation. (iv) Simplified synthesis complexity: The method avoids complex heterostructuring or capping agents. Therefore, it is foreseeable that the BDCA solution method may hold promise in addressing the photocorrosion issue of other types of MS photocatalysts. Building upon the established success of the BDCA solution method in effectively suppressing the photocorrosion issue of CdS, our group subsequently synthesized ZnCdS solid solution photocatalysts employing this methodology [84]. Significantly, we pioneered the novel concept of “controllable-photocorrosion” in this research. This conceptual breakthrough represents a paradigm shift, effectively transforming photocorrosion from a detrimental phenomenon impeding material stability into a strategically leveraged functional attribute. Compared with CdS, ZnS demonstrates markedly superior photostability, attributable to its stronger Zn–S bonding. Nevertheless, its wide bandgap (about 3.4 eV), exceeding even that of TiO_2_, fundamentally constrains visible-light PHE utility [234]. The strategic formation of ZnCdS solid solution effectively addresses this limitation, synergistically combining the visible-light responsiveness of CdS with the durability of ZnS, along with the tunable bandgap (Fig. 16) [300–303]. This alloying approach yields an efficient visible-light photocatalyst for HER, harmonizing tunable bandgap energetics with enhanced photocorrosion resistance [304]. Contemporary reviews have extensively documented these distinctive advantages and the developmental trajectory of ZnCdS in PHE [55, 300]. Notably, such analyses consistently emphasize resolving ZnCdS photocorrosion as a critical research priority. Prevailing mitigation strategies, as cataloged in prior syntheses (Sect. 5.2.1), primarily encompass defensive paradigms: bandgap tuning via Zn/Cd stoichiometric control, morphological optimization, sulfur vacancy engineering, cocatalyst functionalization, and heterojunction construction.

**Fig. 17:**
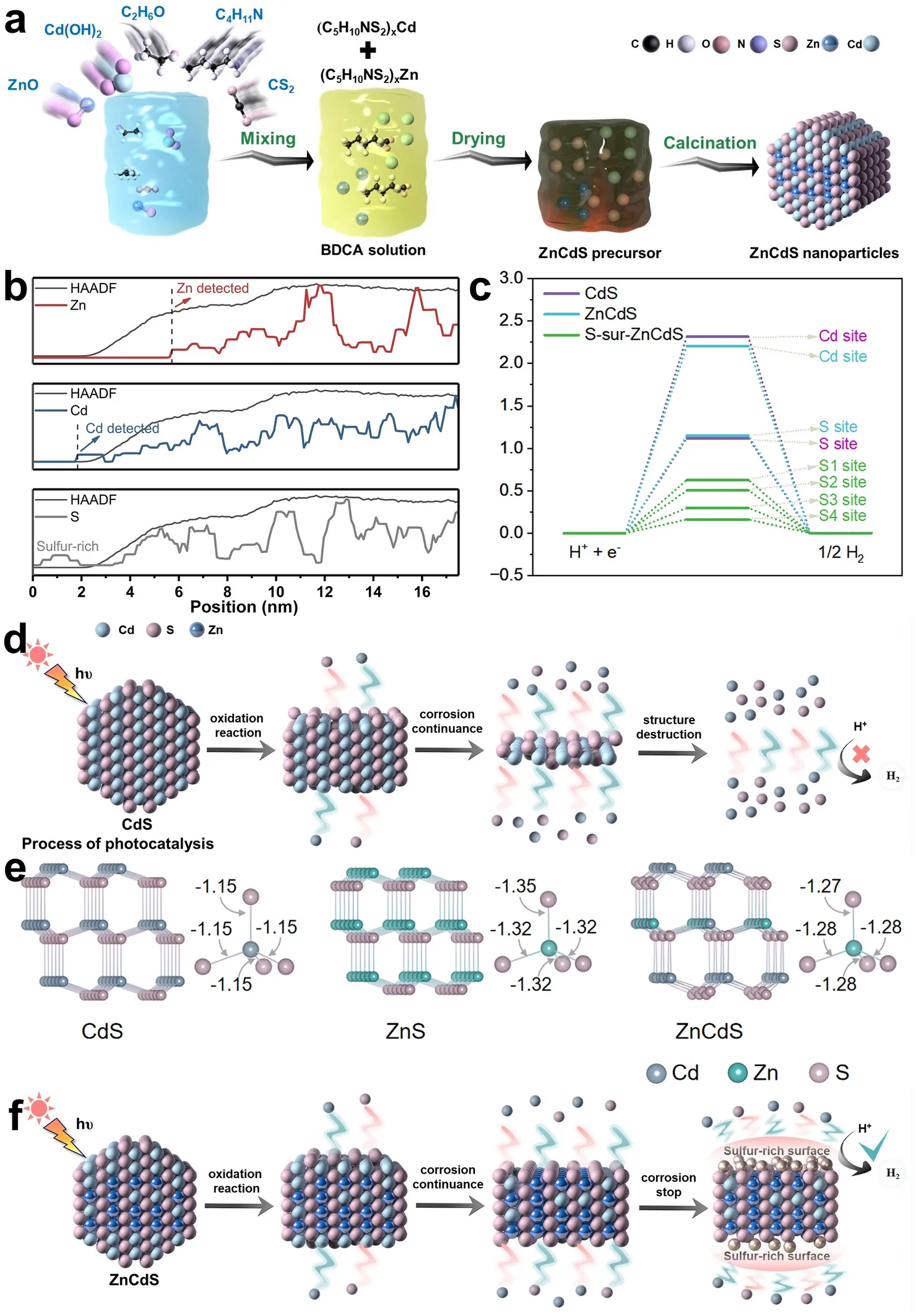
Synthesis and multiscale characterization of BDCA-synthesized ZnCdS solid solution photocatalyst with unique controllable-photocorrosion feature. **a** Schematic illustration of the ZnCdS solid solution synthesis via the BDCA solution process. **b** Compositional line-scan profile acquired by STEM-EDS. **c** Computational evaluation of ΔG_H*_ for CdS, ZnCdS, and sulfur-enriched ZnCdS surfaces (S-sur-ZnCdS). **d** Comparative schematic depicting the photocorrosion pathway in pristine CdS. **e** Bulk structural analysis: Atomic configurations and computed crystal orbital ICOHP values for Cd–S and Zn–S bonds in CdS, ZnS, and ZnCdS. **f** Integrated mechanistic model of the photocorrosion process in BDCA-synthesized ZnCdS: (i) Under illumination, photogenerated holes oxidize the sacrificial sulfur-rich surface and the underlying Cd-rich layer, resulting in the generation of reactive sulfur species. (ii) Photocorrosion progresses inward but is confined to the upper Cd-rich region. (iii) Photocorrosion terminates upon reaching the subsurface Zn–S layer, where stronger Zn–S bonds (evidenced by more negative ICOHP values) act as the kinetic barrier. (iv) Establishing of dynamic equilibrium: sacrificial corrosion continuously regenerates catalytically active sulfur sites at the surface, while the robust Zn–S subsurface preserves structural integrity, leading to self-optimized and stable PHE activity. Reproduced with the permission of Ref. [84] Copyright 2025, Wiley

Building upon the BDCA solution-based methodology for synthesizing CdS (involving the dissolution of Cd(OH)_2_ in BDCA solution), this research extended this methodology to incorporate ZnO dissolved within the BDCA solution, ultimately resulting in the successful fabrication of ZnCdS photocatalyst, possessing a uniquely engineered structure compared with the ZnCdS fabricated by the conventional extra sulfur source method: a sulfur-rich surface layer and a strategically positioned Zn-subsurface distribution (Fig. 17a). HRTEM confirmed the nanoparticle morphology and crystallinity, while high-angle annular dark-field scanning TEM (HAADF-STEM) elemental mapping and STEM-EDS line scanning (Fig. 17b) provide direct evidence of the critical subsurface Zn enrichment beneath the Cd-rich surface. The initial sulfur-rich surface acted as a sacrificial hole scavenger and provides abundant active sites for the HER, which has also been proved by FT-IR in this work. This specific architecture, subsurface Zn and a sacrificial S-rich surface, forms the foundation for controllable-photocorrosion. In detail, contrary to conventional photocorrosion leading to degradation of MS photocatalysts, the BDCA-ZnCdS exhibited increasing PHE activity during extended illumination. The PHE rate surged 2.5-fold over the first five cycles (reaching 30.12 mmol g^−1^ h^−1^) and stabilized thereafter. This phenomenon stems from a self-optimizing mechanism: During the initial PHE, photogenerated holes oxidize the sacrificial sulfur-rich surface and the outermost CdS layer. The photocorrosion generates reactive sulfur species that accumulate in situ, forming a new catalytically active sulfur-rich surface. DFT calculations (Fig. 17c) proved these newly formed sulfur sites (S3: Δ*G*_H*_ = 0.16 eV; S4: Δ*G*_H*_ = 0.30 eV) exhibit near-optimal Δ*G*_H*_, significantly enhancing HER kinetics compared to pristine CdS or ZnCdS surfaces (Δ*G*_H*_ > 1.0 eV). Simultaneously, the strong Zn–S bonds reflected by the ICOHP (ICOHP = − 1.27 to − 1.28 vs. Cd–S ICOHP = − 1.15) act as an intrinsic barrier, confining further corrosion to the Cd-rich upper layers once the Zn-subsurface is reached. This confinement prevents bulk degradation, establishing a dynamic equilibrium between sacrificial corrosion (providing new active S-sites) and structural preservation (enabled by subsurface Zn).

Based on the above results, we redefine the photocorrosion of MS photocatalyst (Fig. 17d–f): uncontrolled photocorrosion rapidly oxidizes S^2−^ and dissolves Cd, degrading CdS, whereas the BDCA-ZnCdS deliberately employs a controlled corrosion process. The initial S-rich surface and subsurface Zn distribution transform a typically detrimental phenomenon into a functional strategy: (i) The initial sulfur-rich layer sacrificially scavenges holes, mitigating hole-induced bulk damage; (ii) the controlled corrosion of the cadmium-rich surface generates highly active in situ S-sites and thus boosts HER activity; (iii) the robust Zn–S subsurface layer terminates corrosion progression, ensuring long-term structural integrity. This controllable-photocorrosion mechanism creates a self-sustaining system where corrosion product (sulfur) becomes the functional site, and corrosion depth is physically limited, resulting in both rising and sustained high activity. It represents a fundamental shift that leveraging photocorrosion productively for enhanced functionality and stability, rather than merely seeking its suppression.

#### Thermodynamic and Kinetic Foundations of Controllable-Photocorrosion

Beyond the above-mentioned phenomena description, the fundamental of controllable-photocorrosion can be also strictly explained through coupled thermodynamic and kinetic analyses. Thermodynamically, the resistance of MS photocatalysts to oxidative destruction is governed by the bond dissociation energy (BDE) of M–S bonds [208]. Representatively, in ZnCdS solid solutions, the Zn–S bond exhibits an obviously higher BDE (2.7 eV) compared to Cd–S (2.3 eV), as also evidenced by ICOHP calculations (− 1.32 of Zn–S and − 1.15 of Cd–S). This obvious energy difference creates a thermodynamic sink that confines the anodic corrosion to the Cd-rich surface layer, while the underlying Zn–S network simultaneously acts as an intrinsic barrier against the bulk destruction of the entire photocatalyst [84]. At the same time, such bond energy gradients can be quantitatively mapped via DFT-based ICOHP analysis, which provides a predictive parameter for designing corrosion-resistant layered architectures. Kinetically, the self-limiting feature of controllable-photocorrosion arises from the dynamic balance between the hole-induced oxidation and in situ passivation. The real-time detecting using in situ Raman spectroscopy and electrochemical impedance spectroscopy reveals that the initial photocorrosion rate of the sulfur-rich surface (*k*_initial-corrosion_ ≈ 10^−3^ s^−1^) decelerates by over an order of magnitude after the formation of reactive-sulfur overlayer (*k*steady-corrosion ≈ 10^−4^ s^−1^) [305]. This kinetic transition is accompanied by a progressive anodic shift in the flat-band potential (≈ + 0.15 V), indicating the increasing of a hole-blocking interfacial dipole [306]. Quantitatively, the photocorrosion inhibition efficiency (*η*_PI_) can be expressed as: *η*_PI_ = (1 – *j*_modified_/*j*_pristine_) × 100%, where *j*_modified_ and *j*_pristine_ are the photocorrosion-current densities of engineered and pristine MS photocatalyst, respectively. Taking the BDCA-ZnCdS as the example, *η*_PI_ exceeds 80% after five PHE cycles, a metric that relates directly with the preservation of PHE activity. Such quantitative parameters, combining bond-energy mapping, photocorrosion rate spectroscopy, and interfacial kinetic profiling, transform the controllable-photocorrosion from a descriptive concept into an advanced design concept rooted in MS photocatalysts’ thermodynamics and reaction dynamics.

#### Quantitative Superiority of BDCA in Photocorrosion Suppression

To quantitatively evaluate the superiority of BDCA solution method in mitigating photocorrosion issue and enhancing PHE performance of MS photocatalyst, we also systematically compare key metrics of defect density, photocorrosion resistance, and photostability between BDCA-synthesized and conventionally synthesized MS photocatalysts (Table 1). As illustrated in Fig. 15d, e, BDCA-synthesized CdS (CdS-500) exhibits a remarkable PHE rate of 7.294 mmol g^−1^ h^−1^ under visible light, surpassing most conventional CdS-based systems. More importantly, it maintains over 95% of its initial activity after 960 min of continuous illumination, demonstrating exceptional photostability. In contrast, conventionally synthesized CdS typically suffers from rapid PHE activity decay (generally > 50% loss within a few hours) due to severe photocorrosion. The enhanced photostability is attributed to the intrinsic sulfur-coordination directionality of the BDCA process, which minimizes S-vacancy defects, a primary initiation site for photocorrosion. DFT analyses (Fig. 15f–i) reveal that BDCA-synthesized CdS possesses a more robust Cd–S bonding environment and favorable ΔG_H*_ at interfacial S-sites, further promoting HER kinetics while resisting oxidative degradation. Similarly, for ZnCdS solid solutions, the BDCA method enables the unique controllable-photocorrosion mechanism (Fig. 17l–n), where a sacrificial sulfur-rich surface and a Zn-enriched subsurface work synergistically to enhance both activity and durability. As also shown in Fig. 17i, BDCA-ZnCdS exhibits a 2.5-fold increase in PHE rate over initial cycles, reaching 30.12 mmol g^−1^ h^−1^, and stabilizes thereafter, a behavior unattainable with conventional extra sulfur source methods.

**Table 1:** Comparative performance metrics of BDCA-synthesized and conventional synthesized MS photocatalysts

Photocatalyst	Synthesis method	Defect density (approximate)	PHE rate (mmol g^−1^ h^−1^)	Stability (Cycles/duration)	Key Observations	References
CdS	BDCA	Low (S-vacancy minimized)	7.294	> 95% after 960 min	High phase purity, robust Cd–S bonds	[299]
CdS	Conventional	High (S-vacancy rich)	About 1–4	< 50% after 4–6 h	Rapid photocorrosion, activity decay	[296]
ZnCdS	BDCA	Moderate (engineered)	30.12	2.5 times increase then stable	Controllable-photocorrosion, self-optimizing surface	[84]
ZnCdS	Conventional	High	About 5–15	Gradual decay	Uncontrolled corrosion, bulk degradation	[300]
CuPbSbS_3_	BDCA	Low (defect-tolerant)	0.2508	~ 82% after 12 h	3D electronic dimensionality, high mobility	[210]

Therefore, the BDCA solution method is indeed a superior synthesis method for suppressing the photocorrosion issue of MS photocatalysts, which has been strictly proved in CdS and ZnCdS. This intrinsic sulfur-coordination directionality synthesis method not only suppresses defect formation and photocorrosion but enables novel corrosion-mediated activation pathways, establishing it as a superior synthesis strategy for developing highly stable and efficient MS photocatalysts.

#### Scope and Fundamental Preconditions of the Controllable-Photocorrosion

The advanced controllable-photocorrosion concept, as exemplified by BDCA-synthesized MS photocatalyst of ZnCdS, represents a paradigm shift from perceiving photocorrosion as a purely detrimental process to harnessing it for the functional enhancement. A critical factor that arises is the generalizability of this strategy across broader the variety of MS photocatalysts. The viability of this concept is not universal but hinges upon the fulfillment of several fundamental material prerequisites, which can be speculated from the mechanistic model established for ZnCdS. First, a sacrificial and dynamically renewable surface layer is essential. The initial sulfur-rich surface in BDCA-ZnCdS acts as the designable hole scavenger. This layer is consumed in a controlled path, generating in situ reactive sulfur species that serve as highly active sites for the HER, as confirmed by their near-optimal ΔG_H*_. For this mechanism to be replicable in other MS systems, the photocatalyst should either possess or be engineered to develop a surface that can undergo a similar transformation, yielding photocorrosion products that are catalytically beneficial rather than passivating. Second, an intrinsic corrosion-inhibiting barrier is also essential. Specifically, the robust Zn–S bonds in the subsurface region of ZnCdS function as a physical and energetic barrier that confines the corrosive process to the upper layer, which prevents the uncontrolled bulk degradation that plagues conventional MS like CdS. Therefore, extending the advanced concept of controllable-photocorrosion requires the MS photocatalyst to have an inherent structural or compositional feature, such as subsurface layer with significantly stronger metal–sulfur bonds, different crystal phase, or stable secondary compound, that can definitively terminate the corrosion front. For instance, while CdS itself lacks such a barrier, engineering a core–shell or gradient structure with a stable ZnS or Sb_2_S_3_ core could potentially introduce one. Conversely, materials like MoS_2_, with their layered van der Waals structures and inherent stability in reducing environments, may not readily form a sacrificial surface layer, presenting a different set of challenges.

Therefore, the advanced concept of controllable-photocorrosion is most promising for MS photocatalysts whose composition and structure can be facilely and precisely engineered to satisfy the dual criteria: a sacrificially active surface and a structurally robust, corrosion-confining subsurface. This advanced design concept opens a new avenue for a wide range of MS photocatalysts where elemental segregation or gradient synthesis can be exploited. Future research should focus on illustrating the thermodynamic and kinetic boundaries of this phenomenon through coupled theoretical and experimental studies, paving the way for its rational deployment beyond ZnCdS.

## Discussion and Outlooks

The development of photocatalysts combining with exceptional semiconductor properties with high photostability is critical for efficient PHE via water splitting. MS photocatalysts with the narrow-bandgaps, enable visible-light-driven PHE, yet research has largely overlooked a fundamental semiconductor characteristic, electronic dimensionality. MS semiconductors with 3D electronic dimensionality offer lower carrier effective mass, high light absorption coefficient, and unique defect tolerance, collectively boosting the concentration of HER-participating photogenerated electrons, thereby enabling a qualitative leap in PHE efficiency. Equally vital, effectively addressing the persistent challenge of photocorrosion in MS photocatalysts is crucial for ensuring their long-term operational stability. Consequently, building upon the overall summarization of the development route of MS photocatalysts, this review provides a comprehensive analysis of advanced design concept centered on harnessing 3D electronic dimensionality and mitigating photocorrosion, especially controllable-photocorrosion. Key research advancements are systematically examined, encompassing the rational design and PHE application of 3D electronic dimensionality materials exemplified by CuPbSbS_3_ and innovative synthesis approaches, notably the intrinsic sulfur-coordination-directed method, designed to fundamentally resolve the photocorrosion issue of MS photocatalyst. To quantitatively benchmark the PHE performance advantages conferred by the advanced design concepts, Table 2 comprehensively compares the PHE performance of BDCA-synthesized MS photocatalysts with other state-of-the-art systems, including single-atom-supported MSs, metal–organic frameworks (MOFs), and covalent organic frameworks (COFs). Notably, while certain single-atom, MOFs, or COFs systems exhibit the higher maximum rates, these often require noble-metal cocatalysts (typically Pt) or represent outcomes of extensive, long-term optimization. In contrast, the BDCA-derived MSs, achieving high performance without noble metals in their early development stage, exhibit immense potential. Their unique combination of 3D electronic dimensionality and controllable-photocorrosion mechanisms provides a foundational platform for the rapid, efficient, and stable PHE, positioning them as the highly promising candidates for scale-up solar fuel production. While commendable initial progress has been achieved through these advanced design concepts, considerable room for enhancement remains to realize the ultimate goal of commercially viable PHE technology. Current challenges and future perspectives based on this design concept mainly include the following aspects:

(i) Prioritizing the low-toxic MMS photocatalysts. Current researches have substantiated that the MMS photocatalysts offered superior compositional tunability compared to conventional BMS photocatalysts. This inherent flexibility facilitates the exploration of superior semiconductor properties. Building upon this foundation, the innovative integration of the electronic dimensionality concept has led to the development of CuPbSbS_3_, a defect-tolerant semiconductor with 3D electronic dimensionality, demonstrating remarkable efficiency in both PHE and Piezo-PD. However, the significant Pb content in CuPbSbS_3_ raises substantial toxicity concerns, presenting critical impediments to its large-scale PHE implementation. As discussed in Sect. 4.1, the representative 3D electronic dimensionality photocatalyst of CsPbI_3_ has been extensively explored for PHE application; substitution with Sn produces non‐toxic CsSnI_3_ but sacrifices 3D connectivity (due to Sn valence instability), degrading photocatalytic efficacy [307–310]. In this case, future research is suggested to extend beyond straightforward elemental substitution. A more profound unresolved issue emerges: Can researchers simultaneously achieve environmental sustainability and superior 3D electronic dimensionality by designing novel crystal architectures or exploring specific elemental combinations? This includes, for instance, stabilizing low- or non-toxic elements with similar lone-pair effects or developing entirely Pb-free 3D chalcogenide frameworks. Addressing this challenge needs a fundamental approach rooted in the principles of the atomic orbital interaction. Continuously, combining advanced theoretical calculation methods with high-throughput screening will be further essential to identify low- or non-toxic elements and their corresponding crystal configurations capable of supporting stable, spatially continuous orbital hybridization across all three dimensions. Therefore, the solution of this pivotal issue represents an indispensable step toward translating high-performance, environmentally benign advanced MS photocatalysts into scale-up applications.
(ii) Development of machine learning (ML) on the design of advanced MS photocatalysts. Advancing photocatalyst design methodologies and deepening the mechanistic comprehension of photocatalytic phenomena are paramount for enhancing photocatalyst efficacy [311–315]. Equally critical is the elucidation of reaction mechanisms and optimization pathways for semiconductor photocatalysts to achieve superior PHE performance. Beyond conventional DFT calculations and experimental refinements, the systematic exploration of materials chemistry space via ML offers a potent strategy for accelerating the discovery and development of advanced semiconductor photocatalysts, a paradigm increasingly validated by contemporary data science research [316–322]. In 2022, Mai et al. highlighted transformative role of ML in advancing photocatalyst discovery, particularly for PHE and PD [323]. Specifically, ML techniques accelerate the screening of complex material spaces by establishing structure–property relationships beyond conventional trial-and-error approaches. For photocatalytic polymers, gradient boosting and random forest models identify critical descriptors like frontier orbital energies and bandgaps, enabling rapid optimization of hydrogen evolution rates. In oxide photocatalysts, ML integrates with DFT and experimental data to optimize bandgap engineering and reaction kinetics in TiO_2_ and perovskites, though data scarcity remains a constraint. Significantly, the analysis of BMS and other 2D/3D materials driven by the ML revealed the pivotal influence of structural dimensionality, governing charge carrier mobility, interfacial electron transfer, and light absorption depth. Therefore, future efforts can prioritize machine models incorporating 3D electronic dimensionality descriptors, focusing on orbital overlap or DOS anisotropy, to enable precise design of advanced MS photocatalysts, along with standardizing high-throughput data generation and extending ML to dynamic reaction mechanisms.
(iii) Diversifying MMS photocatalysts synthesis via BDCA. Current researches have demonstrated that the intrinsic sulfur-coordination directionality BDCA solution method can effectively mitigates the photocorrosion issue in traditional BMS photocatalyst of CdS. Building upon this success, the synthesis was extended to MMS photocatalyst of ZnCdS and established the controllable-photocorrosion mechanism that harnesses the beneficial aspects of this typically detrimental phenomenon. Therefore, the versatile solubility of BDCA solution toward various MO and metal hydroxides (Fig. 14b) theoretically enables the synthesis of diverse MMS photocatalysts. To date, only three kinds of MS photocatalysts of CuPbSbS_3_, CdS, and ZnCdS have been developed for PHE using this approach. Other BMS/MMS materials remain unexplored. Given the intrinsic sulfur-coordination directionality nature of BDCA and the differential solubility of various MO (or metal hydroxides) within it, it is postulated that other BMS/MMS systems fabricated through this route may possess superior band structure characteristics. Consequently, future research should prioritize the diversified synthesis of BMS/MMS photocatalysts using the BDCA solution method, coupled with joint theoretical–experimental studies to elucidate critical properties such as elemental distribution profiles and carrier transport mechanisms. This approach holds dual promise: substantially suppressing photocorrosion while unlocking the distinctive properties inherent to BDCA-synthesized MS photocatalysts, ultimately advancing highly efficient PHE.
(iv) Further widening the advanced synthesis method with intrinsic sulfur-coordination directionality. Existing research has confirmed that the BDCA solution possesses an intrinsic sulfur-coordination directionality property, which significantly suppresses photocorrosion in MS photocatalysts and substantially enhances their long-term photostability. Notably, the formation and preparation of BDCA solution involve a highly exothermic reaction between CS_2_ and C_4_H_11_N, necessitating meticulous dropwise addition at approximately one drop per second to mitigate uncontrolled heat release [194]. This procedural constraint, widely documented in studies on BDCA-assisted MS thin-film deposition, imposes inherent limitations on scalability. Consequently, while the BDCA method offers distinct advantages for photocorrosion mitigation, its practical implementation for large-scale PHE is constrained by the vigorous reaction kinetics and slow preparation protocol. Thus, methodological refinements addressing these synthesis challenges remain imperative to unlock the full potential of BDCA-synthesized MS photocatalysts in commercial applications. For instance, Koskela et al. [191] “alkahest” thiol-amine solvent system dissolves diverse metals and chalcogenides, broadening solution-processing flexibility for MS thin films. Nevertheless, analogous to the highly toxic and hazardous hydrazine-based solution methods, the thiol-based solvent systems exhibit significant toxicity and volatility. These inherent drawbacks substantially impede their scalable adoption for synthesizing advanced MS photocatalysts. Therefore, future research on advanced synthetic methodologies should prioritize the exploration of safer and more efficient solution processes while preserving the critical intrinsic sulfur-coordination directionality property. Such developments would concurrently enhance the mitigation of photocorrosion and the strategic utilization of its beneficial aspects, ultimately advancing the efficiency of PHE systems.
(v) Strategic combination of advanced MS photocatalysts with functional materials. Building upon the rational design of advanced MS photocatalysts, further performance enhancements may be achieved through strategic combination with emerging functional materials such as MOFs, COFs, MXenes, and graphitic carbon nitride (*g*-C_3_N_4_). These materials offer complementary advantages in PHE applications, as demonstrated in various studies: MOFs and COFs provide ultrahigh surface area, tunable pore structures, and well-defined active sites, which facilitate mass transport and adsorption of reaction intermediates [324–331]. MXenes exhibit metallic conductivity, rich surface chemistry, and excellent charge carrier mobility, enabling efficient electron extraction and transfer [332–337]. Meanwhile, *g*-C_3_N_4_ possesses a suitable band structure, high chemical stability, and facile synthesis, making it an attractive component for constructing heterostructures that enhance visible-light absorption and promote charge separation [338–348]. Integrating advanced MS photocatalysts with these functional materials offers a promising pathway toward constructing hierarchical or heterojunction systems with synergistic effects. Such composite architectures could simultaneously enhance light harvesting, improve charge separation efficiency, suppress charge recombination, and increase active sites, collectively contributing to superior PHE performance. In this case, future efforts should focus on the interface engineering, precise band alignment, and understanding the underlying charge transfer mechanisms in these complex systems to unlock their full potential for scale-up PHE applications.
(vi) Focusing on PHE via overall water splitting (PHE-v-OWS). PHE-v-OWS represents the ideal pathway for sustainable hydrogen production, whereas its current efficient realization faces formidable scientific issues [95]. Thermodynamically, PHE-v-OWS is an energetically uphill process (ΔG =  + 237 kJ mol^−1^), requiring the photocatalyst with a bandgap that simultaneously straddles the proton reduction (0 V vs. NHE) and water oxidation potentials (1.23 V vs. NHE). Kinetically, this process is severely hampered by the sluggish OER, a complex 4-hole migration process with inherently high activation barriers and slow reaction rates compared to HER. This kinetic difference often leads to the accumulation of photogenerated holes, accelerating charge recombination and undermining PHE-v-OWS efficiency. A further critical challenge is the existence of dissolved oxygen, which acts as an efficient electron scavenger, generating superoxide radicals that not only compete with proton reduction but initiate detrimental side reactions. At the same time, the most negative factor is the rapid backward reaction, wherein the co-evolved hydrogen and oxygen readily recombine to form water, especially on the surfaces of cocatalysts. This recombination is exacerbated in confined nano-systems and leads to a serious waste of photogenerated charge carriers. Therefore, achieving high PHE-v-OWS efficiency necessitates sophisticated designs for spatial gas separation and meticulous management of reaction microenvironments to suppress these parasitic pathways, making efficient PHE-v-OWS enormously difficult to achieve with current material systems and configurations. The challenges inherent to PHE-v-OWS underscore the necessity for photocatalysts that simultaneously exhibit superior charge transport dynamics and exceptional operational stability. In this case, the strategic construction of 3D electronic dimensionality directly addresses the kinetic limitations of PHE-v-OWS to a large extent. Materials like CuPbSbS_3_, characterized by isotropic band dispersion and high carrier mobility, facilitate the rapid and balanced extraction of both photogenerated electrons and holes to the surface. This is crucial for PHE-v-OWS, where the slow OER kinetics demand efficient hole delivery to active sites. Meanwhile, resolving the pervasive photocorrosion issue of MS photocatalysts is paramount for long-term viability. Advanced design concepts, such as the controllable-photocorrosion mechanism or synthesis methods fostering intrinsic sulfur-coordination directionality, prevent the oxidative degradation of the lattice of MS photocatalysts. When integrated, a photocatalyst endowed with a robust 3D electronic network and corrosion-resistant architecture ensures sustained charge flux for both HER and OER while maintaining structural integrity under prolonged illumination. This synergistic combination of efficient bulk charge transport and durable surface chemistry represents a critical foundational step toward developing viable MS photocatalysts for efficient and stable PHE-v-OWS.
(vii) Toward practical hydrogen production: Natural sunlight and seawater splitting. Although significant research progresses have been achieved in the advanced design of MS photocatalysts, their evaluation mainly performs under the ideal laboratory conditions, using simulated sunlight and pure water with sacrificial reagents. Further, translating these advancements to practical and scale-up PHE needs facing the complexities of real-condition operation: the uncertain spectrum and intensity of natural sunlight and the challenging chemical environment of seawater [349–356]. Specifically, the utilization of natural sunlight introduces the dynamic variables beyond the simulation sunlight of standardized AM 1.5G. The diurnal/seasonal differences in irradiance intensity and spectral composition directly impact the reaction kinetics and stability of PHE [354]. Practical PHE application demonstrations, such as the 100 m^2^ panel reactor array employing SrTiO_3_:Al, demonstrate the engineering challenges and efficiency penalties associated with outdoor operation, where the achieved the solar-to-hydrogen (STH) efficiencies are often sigificantly lower than peak lab-based values [352]. For the advanced MS photocatalysts, whose photocatalytic performance and photostability are sensitive to photon flux and local temperature, designing systems that maintain optimal function under these uncertain conditions is crucial. Strategies may include the adaptive thermal management, harnessing infrared radiation for beneficial temperature control as demonstrated with the InGaN/GaN systems [357], and developing photocatalysts with broad spectral response to the maximize energy harvesting across the solar spectrum. At the same time, direct PHE via seawater splitting (PHE-v-SS) represents a strong yet highly desirable pathway, given the abundance of seawater resources in the Earth [353, 354]. However, the high ionic strength, existence of corrosive chloride ions, and dissolved organic matter generally lead to the photocatalyst poisoning, accelerated photocorrosion of MS, and competitive side reactions. Recent advances have highlighted both the challenges and opportunities of PHE-v-SS. For instance, ions like Na^+^ can enhance interfacial processes or scavenge holes to a certain extent, while others like Mg^2+^ or Ca^2+^ may precipitate with common sacrificial reagents, stabilizing the photocatalytic system [351]. Innovative and advanced photocatalyst design is key to overcoming these issues. Typically, the construction of Schottky junctions with plasmonic metals on vacancy-engineered sulfides has proven effective, not only enhancing charge separation via hot electron injection but forming a protective layer that mitigates chloride-induced corrosion, enabling remarkable PHE rates in simulated seawater [353]. This result proves a move beyond mere corrosion suppression toward the functional integration of protective and photocatalytic components. Therefore, future research direction for MS photocatalysts can bridge the gap between idealized conditions and application-ready performance, which involves several key factors: developing photocatalytic measurement conditions that account for real sunlight variability and complex aqueous matrices like real seawater, moving beyond standard sacrificial reagents systems in pure water; designing the stable composite architectures that synergize 3D electronic dimensionality for efficient charge migration with specifically engineered interfaces, such as corrosion-resistant cocatalysts or selective films, to maintain the photocatalytic performance in the harsh chemical environments and suppress ion poisoning; integrating system-level engineering, including efficient gas separation films and strategies to manage the salinity gradients in seawater, as pioneered in scale-up panel reactors. By coupling advanced design concepts of 3D electronic dimensionality and controllable-photocorrosion with solutions addressing these practical constraints, advanced MS-based photocatalytic systems have great potential to evolving from a promising laboratory phenomenon into a viable technology for sustainable PHE application using natural resources.
(viii) Extending design principles to photocatalytic CO_2_ reduction. In parallel to the advancements in PHE, the advanced design concept of MS photocatalysts emphasizing on 3D electronic dimensionality and controllable-photocorrosion are also demonstrating profound applicability in photocatalytic CO_2_ conversion, opening a new avenue for sustainable fuel and chemical production. The construction of 3D electronic dimensionality, as exemplified in perovskite-inspired systems and tailored MMS photocatalyst, facilitates isotropic charge transport and minimized carrier effective mass, which are equally critical for activating inert CO_2_ molecules and stabilizing multi-electron intermediates required for C–C coupling. Recent breakthroughs in photocatalyst design for photocatalytic CO_2_ reduction, including the Co^0^–Co^δ+^ double-site interfaces for photothermal C–C coupling into light olefins and electron-enriched Bi active sites in BiOCl atomic layers for CO_2_ splitting [358, 359], alongside advancements in electronic restructuring of RuCu alloys for methanol selectivity [360], collectively validate that engineered electronic structures, akin to 3D electronic dimensionality, are essential in guiding the reaction pathways and lowering energy barriers for CO_2_ conversion to multi-carbon products and oxygenates [361]. Moreover, the emerging controllable-photocorrosion paradigm, initially developed for MS-based PHE, also offers a transformative strategy to harness surface dynamics in CO_2_ conversion, where in situ formed sulfur-rich layers or defect-engineered surfaces can act as self-optimizing active sites rather than degradation centers. This synergy between 3D electronic connectivity and controllable-photocorrosion strategies not only addresses the inherent instability of MSs under photo-oxidative conditions but unlocks new reaction pathways of photocatalytic CO_2_ reduction to methanol or long-chain hydrocarbons. Integrating these advanced design concepts into photocatalytic CO_2_ reduction systems can thus accelerate the development of stable, efficient, and scalable MS-based photocatalysts for solar-to-chemical energy conversion, bridging the gap between hydrogen evolution and carbon–neutral synthesis.

**Table 2:** PHE performance comparison between BDCA-synthesized MS photocatalysts and other representative advanced photocatalysts

Category	Specific photocatalysts	Synthesis method	Bandgap (eV)	PHE rate (mmol g^−1^ h^−1^)	Stability (cycles/duration)	Key Observations	References
Advanced design of MS photocatalysts for PHE	CuPbSbS_3_	BDCA	1.38	0.2508	~ 82% after 12 h	3D electronic dimensionality, high mobility	[210]
	CdS	BDCA	~ 2.4	7.294	> 95% after 960 min	High phase purity, robust Cd–S bonds	[299]
	ZnCdS	BDCA	tunable	30.12	2.5 times increase then stable	Controllable-photocorrosion, self-optimizing surface	[84]
SA^a)^ supported MS photocatalysts	Ni-SA/ZnIn_2_S_4_	Electrostatic adsorption	~ 2.5	1.788	6 cycles no decay	Ni-SA anchored on S-vacancy enriched surface; Ni acts as electron trapping center	[362]
	CoP-SA-*V*_P_^b)^/Cd_0.5_Zn_0.5_S	Hydrothermal & phosphorylation	~ 2.41	68.33	5 cycles (30 h), negligible decay	P-SA vacancies boost charge separation	[363]
	Co-SA/CdS	In situ photochemical anchoring	~ 2.4	60.10	Stable over 12 h; robust HER durability	Co-SA anchored at *V*_Cd_; enhanced H_2_O dissociation	[364]
	Pt-SA/CdIn_2_S_4_-*V*	Impregnation & annealing	~ 2.16	0.8272	4 cycles stable	Synergy of Pt-SA and *V*_S_ enhances charge separation	[365]
	Rh-SA-@MoS_2_/ZnCdS-*V*_S_	Hydrothermal & low-temp calcination	2.3	39.827	Slight decay after cycling	Rh-SA & *V*_S_ synergistically promote charge separation	[366]
	Rh-SA-ZnCdS-*V*_S_	Hydrothermal & low-temp calcination	~ 2.3	30.512	~ 82% after 4 cycles	Rh SA & *V*_S_ synergistically promote charge separation	[367]
MOF-based photocatalysts	MoO_3_/MIL-125-NH_2_	Impregnation	2.65 (MOF)	0.399	5 cycles no decay	First demonstration of band bending in MOFs	[368]
	Pt-SA@Pd-PCN-222-NH_2_	Precoordination confinement	~ 1.96	16.59	7 cycles stable	Synergy of Pt-SA and Pd-porphyrin; high atom utilization	[369]
	COK-47 (Ti-MOF)	Microwave-assisted solvothermal	2.85	4.3	Structure preserved after 14 h irradiation	2D SBU^c)^ promotes charge separation, LMCT^d)^ mechanism	[370]
	Au@NH_2_-UiO-66/CdS	Self-assembly growth	Visible-light responsive	0.6649	6 cycles stable	LSPR^e)^-induced hot electron transfer prolongs carrier lifetime	[371]
	Fe_0.25_Ni_1.75_DMBD^f)^-NS@CdS	Hydrothermal	1.35 (MOF) 2.41 (CdS)	12.15	3 cycles no decay	Fe/Ni bimetallic synergy, heterojunction-enhanced charge separation	[372]
	C_60_@NU-901^ g)^	Adsorption encapsulation	~ 1.8	22.3	16 h continuous illumination	Host–guest enhanced built-in electric field	[373]
	HE-MOF-NS^h)^	Solvothermal	2.37	13.24	Stable over 4 cycles	High-entropy MOF nanosheets and *p*-type, stable	[374]
	Trimetallic MOF (Cd/Ni/Ho)	One-pot solvothermal by sulfurization	~ 2.41	40.06	20 h	Synergy of uniformly dispersed photosensitive photothermal sites	[375]
	MIP-209(Ti-Cr)-NO_2_^i)^	Green solvothermal	3.34	1.1624	4 cycles retained activity	Ti_12_ oxo-cluster, Cr-doped, no noble-metal cocatalyst	[376]
	UiO-66-NM^j)^	Post-synthetic modification	-	0.381	Crystallinity retained after 5 cycles	NAD(P)H-mimicking enhances PCET^k)^ for hydrogen evolution	[377]
	Cu–BCA–Hf–O (MOF)	Stepwise solvothermal	1.42	49.83	No obvious decrease in 5 cycles	Record high activity among pristine MOF, ultra-stable framework	[378]
MOF-based photocatalysts	Sp^2^-C-linked triazine-based COF	Gradient heating strategy	1.95	107.38 (with 12 wt% Pt)	89.4% after 5 cycles	Sp^2^–C linkage enhances π-delocalization and charge transfer	[379]
	COF-OH-3	Solvothermal	2.28	9.89	30 h stable in 6 cycles	Proton tautomerism tunes band structure	[380]
	Pt clusters on PY-DHBD-COF	In situ photodeposition	2.28	42.432	60 h no significant decay	Uniform Pt clusters via hydroxyl/imine sites	[381]
	TpPa-Cu(II)-COF^m)^	Solvothermal synthesis & Cu(II) coordination	~ 2.0	14.72	Stable for 24 h irradiation	Enantioselective combination enhances hole’s extraction	[382]
	Ni-COF-SCAU-1	Acid-catalyzed Schiff reaction	~ 1.99	197.46 (with 3% Pt)	Excellent stability during reaction	Ni intercalation enhances exciton dissociation and charge transfer	[383]
	Co/Zn-Salen-COF	Schiff-base condensation & metalation	~ 2.45	1.378	Sustained activity over 20 h	Salen-Co sites in COF framework enable efficient charge transfer	[384]
	TpBpy-Ni2% (Ni-COF)	Solvothermal coordination	1.84	51.3	12 cycles (48 h)	Panchromatic response and efficient MLCT	[385]
	Enaminone-linked COF	Solvothermal method	–	2.396	4 cycles negligible drop	Superior exciton dissociation and high chemical stability	[386]
	Cobaloxime-integrated COF	Covalent click reaction	~ 1.74–1.95	19	4 cycles stable	Alcohols act as hole scavengers, enhancing charge separation	[387]
	TCDA^o)^-COF	Schiff-base condensation	2.12	23.6	5 cycles ~ 70% activity retained	Partially conjugated linkage; D-π-A structure; High surface area	[388]
	COF-954 (Pt co-catalyst)	Schiff-base condensation	1.97	137.23	Stable over 20 h continuous test	Kagome lattice enhances charge separation	[389]
	Nano-COF	Surfactant-assisted aqueous synthesis	~ 2.99–2.90	392.0	Sustained H_2_ production up to 42 h (rate decline after 20 h)	Nanoscale COFs with enhanced light harvesting and reverse concentration dependence	[390]

a) *SA* singe atom, b) *V*: vacancies (defects), c) *SBU*: secondary building unit, d) *LMCT* ligand to metal charge transfer, e) *LSPR* localized surface plasmon resonance; f) *DMBD* 2,5-dimercapto-1,4-benzenedicarboxylic acid; g) *NU-901* nano-sized zirconium-based MOF, h) *HE-MOF-NS* high-entropy MOF nanosheets, i) *MIP*: materials from institute of porous materials of Paris, j) *NM* nicotinic acid and 3-carboxy-1-methylpyridinium iodide, k) *PCET* proton-coupled electron transfer, l) *PY-DHBD-COF* an adjacent hydroxyl group and imine bond in each constitutional unit (PY: 1,3,6,8-tetra(4-formylphenyl)pyrene and DHBD: 1,4-dihydroxybenzidine), m) *TpPa-Cu(II)-COF* containing phenyl as linker applying as a representative to investigate the coordination structure of the photocatalyst; n) *Ni-COF-SCAU-1* Ni-intercalated fluorenone-based COF, o) *TCDA* three-component donor-π-acceptor
